# Regional citrate anticoagulation for replacement therapy in patients with liver failure: A systematic review and meta-analysis

**DOI:** 10.3389/fnut.2023.1031796

**Published:** 2023-02-16

**Authors:** Bo Peng, Jiaqi Lu, Hebing Guo, Jingyuan Liu, Ang Li

**Affiliations:** ^1^Beijing Ditan Hospital, Capital Medical University, Beijing, China; ^2^Beijing Fengtai Hospital, Beijing, China

**Keywords:** anticoagulation, citrate, liver failure, extracorporeal organ support, renal replacement therapy, molecular adsorbent recirculation system, plasma exchange

## Abstract

**Background:**

Citrate refers to an anticoagulant agent commonly used in extracorporeal organ support. Its application is limited in patients with liver failure (LF) due to the increased risk of citrate accumulation induced by liver metabolic dysfunction. This systematic review aims to assess the efficacy and safety of regional citrate anticoagulation in extracorporeal circulation for patients with liver failure.

**Methods:**

PubMed, Web of Science, Embase, and Cochrane Library were searched. Studies regarding extracorporeal organ support therapy for LF were included to assess the efficacy and safety of regional citrate anticoagulation. Methodological quality of included studies were assessed using the Methodological Index for Non-randomized Studies (MINORS). Meta-analysis was performed using R software (version 4.2.0).

**Results:**

There were 19 eligible studies included, involving 1026 participants. Random-effect model showed an in-hospital mortality of 42.2% [95%CI (27.2, 57.9)] in LF patients receiving extracorporeal organ support. The during-treatment incidence of filter coagulation, citrate accumulation, and bleeding were 4.4% [95%CI (1.6-8.3)], 6.7% [95%CI (1.5-14.4)], and 5.0% [95%CI (1.9-9.3)], respectively. The total bilirubin(TBIL), alanine transaminase (ALT), aspartate transaminase(AST), serum creatinine(SCr), blood urea nitrogen(BUN), and lactate(LA) decreased, compared with those before the treatment, and the total calcium/ionized calcium ratio, platelet(PLT), activated partial thromboplastin time(APTT), serum potential of hydrogen(pH), buffer base(BB), and base excess(BE) increased.

**Conclusion:**

Regional citrate anticoagulation might be effective and safe in LF extracorporeal organ support. Closely monitoring and timely adjusting during the process could reduce the risk for complications. More prospective clinical trials of considerable quality are needed to further support our findings.

**Systematic review registration:**

https://www.crd.york.ac.uk/prospero/, identifier CRD42022337767.

## 1. Introduction

Liver failure (LF) is a critical syndrome commonly seen in clinical practice. Liver failure is a severe liver damage caused by a variety of factors, leading to severe impairment or decompensation of synthetic, detoxification, metabolic and biotransformation functions, and appears as a group of clinical syndromes with jaundice, coagulopathy, hepatorenal syndrome, hepatic encephalopathy, ascites and other major manifestations (1). Studies have demonstrated that LF patients are susceptible to acute kidney injury (AKI) that significantly compromises their prognosis (2). A study by Tujios et al. (3), have found that the morbidity of AKI is approximately 70% in LF patients. Multiple organ failure (MOF) is one of the leading causes of death in critical patients, and extracorporeal organ support (ECOS) could be applied for MOF (4). In the past decades, multiple artificial organ support systems have been applied in the treatment of hepatic and renal failure, such as renal replacement therapy (RRT), molecular adsorbent recirculation system (MARS), and plasma exchange (PE). Extracorporeal blood anticoagulation is the prerequisite for the normal functioning of these systems.

Various anticoagulation strategies have been tried in clinical practice over the years (5). Regional citrate anticoagulation (RCA) is clinically applied since the 1980s (6), and has been receiving increasing attentions. It performs the anticoagulant effect in extracorporeal circulation via chelating calcium ions that present to be a key factor in coagulation cascades (7). As a regional anticoagulation strategy, RCA neither affect the systemic pro-coagulation state, nor disturb the fragile balance of pro- and anti-coagulation in LF patients, which makes it a preferable option for LF patients receiving ECOS (8). Multiple studies have revealed that RCA would be significantly beneficial for prolonging the ECOS lifespan and reducing the risk for hemorrhagic complications (9, 10). Several studies indicate the RCA is safe and effective for RRT in patients with hypohepatia (11–13), especially for continuous renal replacement therapy (CRRT) in which RCA has been recommended as the most proper agent for regional anticoagulation (14). It is safe even for those with severe liver dysfunction (15).

However, the use of RCA in ECOS for LF patients remains controversial. Citrate is physiologically degraded, by the mitochondria of hepatic cells, skeletal muscle cells, and glomerular epithelial cells, into bicarbonate (16). Therefore, LF patients have increased risk of citrate accumulation during RCA-involved ECOS, compared with those un-concomitant with liver dysfunction. Citrate accumulation is characterized by internal environment disturbance, such as hypocalcemia, metabolic acidosis or alkalosis, and Ca_tot_/Ca_ion_ ratio over 2.5 (17). Chelation of citrate with serum calcium ions forms calcium citrate, a soluble complex difficult to be disintegrated, which decreases the serum free calcium so that could induce muscle spasm even cardiac dysfunction (18). Anther common complication of citrate accumulation is metabolic disorder, while the reabsorption of intestinal ammonia would be enhanced in LF patients with metabolic alkalosis leading to severe hyperammonemia so that induces or aggravates hepatic encephalopathy (19). Study by Link et al., (17) has shown that citrate accumulation is associated with decreased liver detoxification function, and is an independent risk factor for 28-day mortality. The Kidney Disease Improving Global Outcomes (KDIGO) lists severe LF as one of a contraindication for citrate-involved anticoagulation (20).

There remain controversies in previous articles on the feasibility of RCA for LF. Therefore, we have summarized RCA in multiple types of ECOS, and have systematically reviewed the efficacy and safety of RCA for the treatment of LF. We expect our findings could provide significant reference for the use of RCA in ECOS. The types of ECOS on which this study mainly focuses are RRT, PE, and MARS.

## 2. Materials and methods

This systematic review and meta-analysis is conducted and reported in accordance with the *Preferred Reporting Items for Systematic Reviews and Meta-Analyses* (PRISMA2020) Statement (21), and the study protocol has been registered on PROSPERO (registration No. CRD42022337767). Ethical approval is not required in that this is a review of published studies.

### 2.1. Search strategy

PubMed, Web of Science, Embase, and Cochrane Library were searched, from January 1st, 2000 to March 8th, 2022, for studies regarding RCA-involved extracorporeal organ support for LF. Each database had specific search strategy, and the search items mainly included “anticoagulation”, “citrate”, “liver failure”, “renal replacement therapy”, “molecular adsorbent recirculation system” and “plasma exchange”. The search strategy was adjusted for different databases. Literature search was conducted by two reviewers independently, and disagreements were settled by a third reviewer. The search items were designed based on the combination of medical subject headings and free words. The author information and reference lists of retrieved articles were manually searched for more study information. We had only included published studies, and no language restriction was set.

### 2.2. Inclusion and exclusion criteria

Study meeting the following criteria would be included:

➀ Case-control study, cohort study, randomized controlled trial (RCT), or single-arm clinical trial.

➁ Patients were diagnosed with LF, including acute LF (ALF) and chronic LF (CLF). ALF is defined as a sudden loss of liver function with no preexistent liver diseases, such as disturbance of consciousness (encephalopathy) and coagulation disorder (typically INR>1.5, not receiving anticoagulants) (22). CLF refers to decompensated liver cirrhosis primarily characterized by ascites, portal hypertension, and hepatic encephalopathy (23).

➂ Participants were aged over 18.

➃ All patients had received RCA-involved ECOS.

➄ Outcome measures included at least one of the follows: filter lifespan, filter blood coagulation, patients‘ survival, incidence of citrate accumulation, incidence of bleeding, changes in liver and renal function indicators, and results of arterial blood gas analysis.

➅ Reported and published in English.

Exclusion criteria were as follows:

➀ Repeated publication.

➁ Other types of study such as animal study, basic research, case-report, literature review, systematic review, letter, conference summary, and study with irrelevant participants or interventions.

➂ Full-text unavailable.

➃ Irrelevant to the efficacy and safety of RCA for LF.

➄ Sample size less than 5.

➅ Data incomplete, missing, or unextractable.

➆ Reported and published in non-English.

### 2.3. Data extraction

Data extraction was conducted by two reviewers independently. All standards and required data were identified before study selection and data extraction to ensure the extraction consistency between the two reviewers. Any discrepancy would be settled by the third reviewer. Titles and abstracts of retrieved articles were browsed to preliminarily identify potential eligible studies, and the full-texts of remaining articles were read to identify studies to be included. The data extracted included name of the first author, nationality, study-design, inclusion and exclusion criteria, publication date, study periods, sample size, characteristics of participants, interventions, filters per capita using, outcome measures (incidence of citrate accumulation, filter lifespan, and changes in blood calcium concentration, electrolytes, and liver and renal function indicators), patients‘ survival, and complications. Primary outcome was the efficacy, assessed via filter lifespan, filter coagulation, and patients‘ survival. Second outcome was the safety, assessed by types and incidence of adverse events, incidence of citrate accumulation and bleeding, and changes in liver function, renal function, and acid-base indicators.

### 2.4. Quality assessment

Methodological quality of included studies were assessed using the Methodological Index for Non-randomized Studies (MINORS) (24), and this process was conducted by two reviewers independently. MINORS has 12 items, and each item can be scored for 0 to 2 indicating on report, incomplete report, and sufficient report, respectively. The first 8 items can be scored for a maximum of 16, and a total score of 24 after adding the latter 4 items. The specific MINORS items are as follows: (1) a stated aim of the study, (2) inclusion of consecutive patients, (3) prospective collection of data, (4) endpoint appropriate to the study aim, (5) unbiased evaluation of endpoints, (6) follow-up period appropriate to the major endpoint, (7) loss to follow-up not exceeding 5%, and (8) prospective calculation of the sample size. The latter 4 items are designed for studies that have control group, which are (1) a control group having the gold standard intervention, (2) contemporary groups, (3) baseline equivalence of groups, and (4) statistical analyses adapted to the study design. In this meta-analysis, non-comparative studies scored for less than 8, within 9 to 12, and over 13 would be considered of low, medium, and high quality, respectively. Comparative studies scored for less than 12, within 13 to 18, and over 19 would be considered of low, medium, and high quality, respectively.

### 2.5. Statistical analysis

All data analyses were performed using R software (version 3.3.3, The R Foundation for Statistical Computing, Austria, Vienna). For continuous variables, the median (IRQ) was transformed in to mean difference (MD) ± standard deviation (SD). For classification variables, the incidence was calculated by dividing the total number of patients by the number of events observed. Fixed-effect model and random-effect model were applied for meta-analysis to pool the effect size (ES) and 95% confidence interval (95%CI). Heterogeneity among included studies was assessed using *I*^2^ statistic. Generally, an *I*^2^<25% indicates low heterogeneity, 25% < *I^2^* < 50% indicates medium heterogeneity, and an *I*^2^>50% indicates significant heterogeneity. Random-effect model would be applied if *I*^2^>50%, otherwise, fixed-effect model would be used (25). Subgroup analysis would be performed if there were sufficient data, to identify the potential sources of heterogeneity. Sensitivity analysis would be conducted if necessary to assess the effect of individual study on the pooled size. Publication bias was assessed using funnel plot, Egger‘s test and Begg’s test. Descriptive statistics was applied to analyze characteristics of the participants. A *p* value less than 0.05 indicated statistical significance.

## 3. Results

### 3.1. Study selection

**FIGURE 1:**
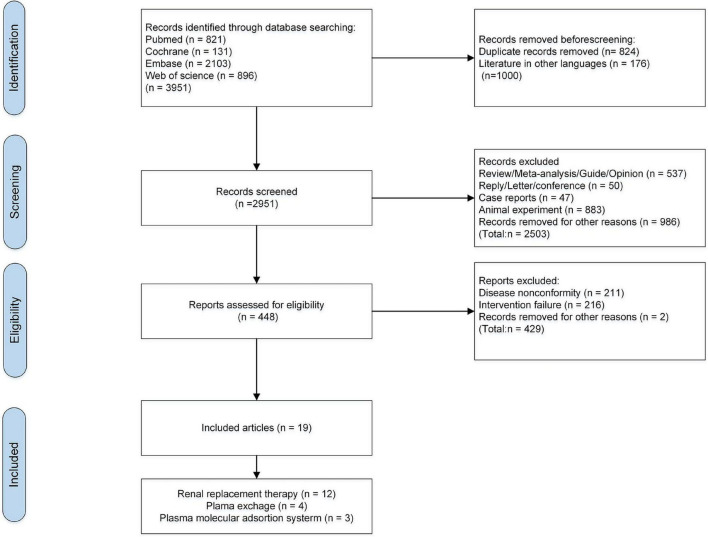
Flow diagram of search results according to PRISMA.

There were 3,951 relevant articles retrieved (821 from PubMed, 896 from WOS, 2103 from Embase, and 131 from Cochrane Library). These articles were screened according to the inclusion and exclusion criteria. There were 824 duplicates and 176 non-English articles removed, and 2,951 remained. After browsing the study-design, titles and abstracts, 2,503 irrelevant studies, 883 animal studies, 47 case-reports, 537 reviews, 50 reviews and meta-analyses, 50 letters and conference summaries were excluded, and 986 studies were removed for other reasons (incomplete and missing data, studies of pathological mechanisms, studies on infants and children, and incomplete full-texts). Then, 448 articles remained for full-text reading, and 429 were excluded. Finally, 19 eligible studies were included (11–13, 26–41). Study selection process are shown in Figure 1.

### 3.2. Characteristics of included studies

There were 19 eligible studies included in this systematic review and meta-analysis, involving 1,026 participants. Among the included studies, 780 patients of 12 studies had received RCA during RRT, 158 patients of 3 studies received RCA during PE, 79 patients of another 3 studies during MARS. Additionally, 9 patients of 1 study had received RCA during RRT-PE combination treatment. Publication date of these studies ranged from 2005 to 2021, with the sample size varying greatly. Only 2 studies recruited over 100 participants. All included studies were observational design, with 8 prospective studies and 11 retrospective studies. As for the nationality, 7 studies were conducted in German, 3 were in China, and the other 9 were in Italy, France, The United States, Brazil, Turkey, India, Belgium, Austria, and Japan, respectively. All these studies were single-centric. Various geographical regions and intervention regimens might be the source of heterogeneity. The primary tool these studies applied for LF severity assessment was the Model for End-Stage Liver Disease (MELD), and Acute Physiology and Chronic Health Evaluation (APACHE-II) and Sequential Organ Failure Assessment (SOFA) were applied to assess the severity of critical patients. Among the 13 RRT-involved studies, CRRT accounted for nearly 80%. Characteristics of included studies are presented in Table 1.

**TABLE 1:** Characteristics of included studies.

References	Country	Study design	Type	Number of patients	Age (years)	Gender (F/M)	MELD score	APACHE II	SOFA score
Yu et al. (26)	China	Prospective	RRT	145	59 (48-72)	53/92	NR	28 (23-35)	NR
Slowinski et al. (13)	Germany	Prospective	RRT	85	63 ± 15	25/60	NR	NR	14 ± 3
Lahmer et al. (12)	Germany	Prospective	RRT	24	59 (29-73)	4/20	35 (25-40)	33 (24-40)	16 (8-19)
De Vico et al. (27)	Italy	Prospective	RRT	15	67.4 ± 11.9	5/10	NR	NR	NR
Schultheiß et al. (11)	Germany	Prospective	RRT	28	57 ± 11	8/20	36 ± 8.8	NR	NR
Pourcine et al. (28)	France	Retrospective	RRT	41	67 (57-73)	19/22	27 (18-42)	NR	NR
Rhee et al. (29)	United States	Retrospective	RRT	32	58.2 ± 14.4	9/23	NR	NR	14.9 ± 3.3
Yu et al. (30)	China	Retrospective	RRT	41	52.49 ± 15.62	22/41	29.78 ± 6.93	17.12 ± 4.30	11.44 ± 4.04
Klingele et al. (41)	Germany	Retrospective	RRT	69	59.1 ± 12.4	22/47	19.7 ± 9.6	NR	NR
Sponholz et al. (31)	Germany	Retrospective	RRT	89	54 (46.5-59.5)	31/58	NR	23 (19-30)	NR
Saner et al. (32)	Germany	Retrospective	RRT	68	47.1 ± 11.8	28/40	NR	17.7 ± 3.7	23.1 ± 9.1
Durão et al. (33)	Brazil	Retrospective	RRT	143	66 ± 16	59/84	NR	27.3 ± 9.3	NR
Ma et al. (34)	China	Prospective	PE	54	50.0 ± 11.3	13/41	NR	NR	NR
Berber et al. (35)	Turkey	Retrospective	PE	59	42 (22-84)	29/30	NR	NR	NR
Maheshwari et al. (36)	India	Retrospective	PE	45	38 (27-68)	14/31	NR	NR	NR
Meijers et al. (37)	Belgium	Prospective	MARS	10	55 ± 10	NR	NR	NR	NR
Falkensteiner et al. (38)	Germany	Retrospective	MARS	49	59 (45.5-64.0)	18/31	32 (18.0-38.0)	24 (20.0-30.0)	11 (8-13.5)
Faybik et al. (39)	Austria	Retrospective	MARS	20	43.5 (23.5-56.5)	NR	31 (26.5-40)	NR	14.2 (4-20)
Yonekawa et al. (40)	Japan	Prospective	RRT + PE	9	65 (54-78)	2/7	NR	NR	NR

RRT, renal replacement therapy; MARS, molecular adsorbent recirculation system; PE, plasma exchange; NR, not reported; MELD, model for end-stage liver disease; APACHE, acute physiology and chronic health evaluation; SOFA, Sequential Organ Failure Assessment.

### 3.3. Quality assessment

Methodological quality of included studies was assessed using MINORS, and the scores are shown in Table 2. There were 3 non-comparative studies assessed via the 8 items, and the other 16 comparative studies assessed via the 4 items. According to different grading standards, 11 studies were graded as low risk of bias, and the total scores indicated high quality. Another 8 studies were graded as medium quality.

**TABLE 2:** Risk of bias in each study assessed using the MINORS tool.

References	Item 1	Item 2	Item 3	Item 4	Item 5	Item 6	Item 7	Item 8	Item 9	Item 10	Item 11	Item 12	Total score
Yu et al. (26)	2	2	1	2	2	2	2	0	2	2	2	1	20
Slowinski et al. (13)	2	2	2	2	2	2	2	0	2	2	2	1	21
Lahmer et al. (12)	2	1	2	2	2	2	2	0	—	—	—	—	13
De Vico et al. (27)	2	1	2	2	2	2	2	0	1	2	1	1	18
Schultheiß et al. (11)	2	1	1	2	2	2	2	0	—	—	—	—	12
Pourcine et al. (28)	2	1	1	2	2	2	2	0	1	2	1	1	17
Rhee et al. (29)	2	1	0	2	1	2	2	0	2	2	2	1	17
Yu et al. (30)	2	1	1	2	1	2	2	0	2	2	2	2	19
Klingele et al. (41)	2	2	2	2	2	2	2	0	2	2	2	1	21
Sponholz et al. (31)	2	2	2	2	2	2	2	0	2	2	2	1	21
Saner et al. (32)	2	2	2	2	2	2	2	0	2	2	2	1	21
Durão et al. (33)	2	2	1	2	2	2	2	0	1	2	1	1	18
Ma et al. (34)	2	2	2	2	2	2	2	0	2	2	2	2	22
Berber et al. (35)	2	2	1	2	1	2	2	0	2	2	2	1	19
Maheshwari et al. (36)	2	1	2	2	1	2	2	0	1	2	1	1	17
Meijers et al. (37)	2	0	1	2	2	2	2	0	2	2	2	1	18
Falkensteiner et al. (38)	2	1	2	2	1	2	2	0	2	2	2	1	19
Faybik et al. (39)	2	1	2	2	1	2	2	0	2	2	2	1	19
Yonekawa et al. (40)	2	0	1	2	1	2	2	0	—	—	—	—	10

Item 1, a stated aim of the study; Item 2, inclusion of consecutive patients; Item 3, prospective collection of data; Item 4, endpoint appropriate to the study aim; Item 5, unbiased evaluation of endpoints; Item 6, follow-up period appropriate to the major endpoint; Item 7, loss to follow-up not exceeding 5%; Item 8, prospective calculation of the sample size; Item 9, a control group having the gold standard intervention; Item 10, contemporary groups; Item 11, baseline equivalence of groups; Item 12, statistical analyses adapted to the study design. Green represents 2 points; Yellow represents 1 point; and Red represents 0 point.

### 3.4. Study results

#### 3.4.1. Efficacy

Efficacy of RCA for LF was assessed according to the mortality, filter lifespan, and filter coagulation. The primary ECOSs in this study included RRT, PE, and MARS.

#### 3.4.2. Mortality

**FIGURE 2:**
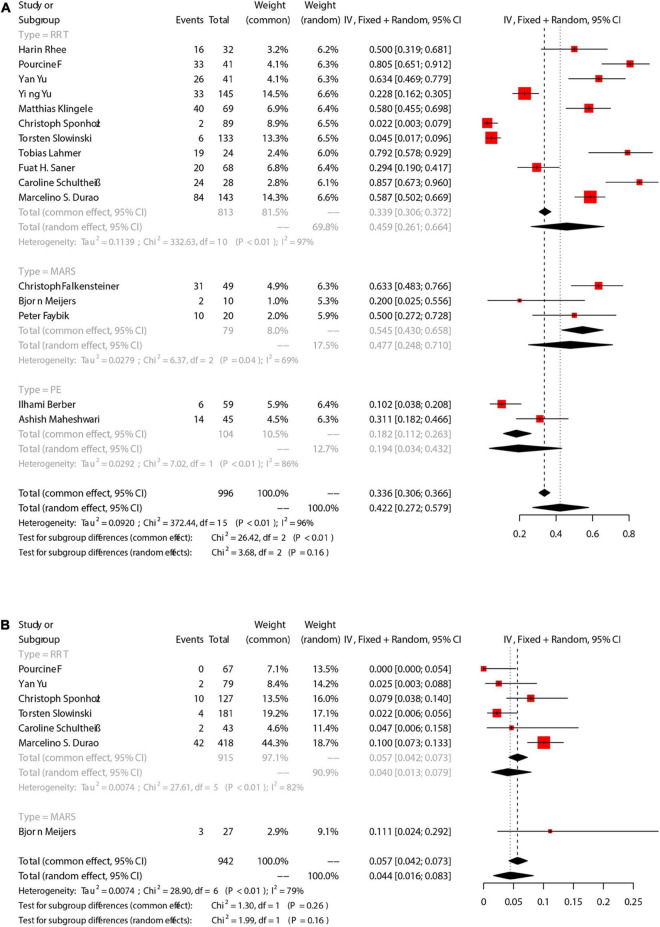
The pooled rates (95% CI) of patient death **(A)** and filter clotting **(B)**. RRT, renal replacement therapy; MARS, molecular adsorbent recirculation system; PE, plasma exchange; CI, confidence interval.

There were 16 studies that reported mortality, and the mortality endpoint subjected to the in-hospital mortality. The overall mortality was 42.2% [95%CI (27.2, 57.9)] (Figure 2A). Mortality in RRT, PE, and MARS group were 45.9% [95%CI (26.1, 66.4)], 19.4% [95%CI (3.4, 43.2)], and 47.7% [95%CI (24.8, 71.0)], respectively. No statistical difference in the mortality among the 3 groups (Chi2 = 3.68, df = 2, *p* = 0.16), while significant heterogeneity was observed (Chi2 = 372.44, df = 15, *P* < 0.01, I2 = 96%).

#### 3.4.3. Filter lifespan

Characteristics of different interventions are shown in Table 3. There were 16 studies that reported the number of times of loop filter, so that the per capita use could be calculated. There are 946 participants involved in the 16 studies, and 3340 sets of loop filter were used (3.5 per capita use). The 11 RRT-involved studies, with 754 patients, had used 2715 sets (3.6 per capita use), and the 3 MARS-involved studies, with 79 patients, had used 179 sets (2.3 per capita use). In addition, 2 studies reported a per capita filter use of 3.9 (446/113) during PE. Among the included studies, 9 reported the average filter lifespan of 37.5 hours. Average filter lifespan was 44.2 h during RRT, 14 during MARS, and not reported during PE. Multiple factors could induce the discontinuation of ECOS such as filter coagulation, Ca_tot_/Ca_ion_ ≥ 2.5, emergency surgery, hemorrhage, machine dysfunction, and clinical decision to discontinue. This would explain the different filter lifespan among individuals.

**TABLE 3:** Characteristics of different treatment patterns.

References	Study timing	Treatment patterns	Number of filters	Filter usage per capita	Filter lifespan	Study subjects	Exclusion criteria
Yu et al. (26)	from January 2014 to August 2015	CRRT	275	275/145	47.50 (37.75-58.58)	LF	CRRT for less than 24 h; any cases with missing data
Slowinski et al. (13)	from January 2008 to February 2010	CRRT	181	181/133	NR	LF	Former use of RCA within 72 h before the study start; participation in other trials
Lahmer et al. (12)	NR	RRT	43	43/24	NR	Decompensated liver cirrhosis or acute liver failure	pH > 7.55 or pH < 7.1; serum ionized calcium < 0.9 mmol/L
De Vico et al. (27)	NR	CRRT	NR	NR	49.76 ± 22.10	LF	NR
Schultheiß et al. (11)	From October 2009 and October 2011	CRRT	43	43/28	NR	Acute LF	pH > 7.55 or < 7.1; Ca_ion_ < 0.9 mmol/l
Pourcine et al. (28)	From January 2014 to June 2015	RRT	67	67/41	NR	Acute or chronic liver dysfunction	Patients with end stage Renal disease
Rhee et al. (29)	From January 1 to December 31, 2017	CRRT	104	104/32	26.0 (14.0-51.0)	Liver disease	CRRT for less than 24 h; CRRT with missing data
Yu et al. (30)	From January 2013 to October 2016	CRRT	79	79/41	NR	LF	Age < 18 years; patients underwent LMWH anticoagulation; patients underwent heparin anticoagulation; patients with severe hyperlacticaemia
Klingele et al. (41)	From January 2009 to November 2012	CRRT	1339	1339/69	62.2 ± 11.2	Severe liver dysfunction	ICG-PDR not performed
Sponholz et al. (31)	From January 2007 to December 2011	RRT	127	127/89	29 (11.0-68.0)	Severe liver dysfunction	NR
Saner et al. (32)	From November 2004 to August 2007	CRRT	NR	NR	22.7 ± 14.6	LF	Age < 18 years; incomplete patient records
Durão et al. (33)	From February 2004 to July 2006	RRT	418	418/143	72 ± 22.2	LF	NR
Ma et al. (34)	From March 2017 to June 2017	PE	203	203/54	NR	HBV-ACLF	Drug-induced liver injury, immune-related liver disease, alcoholic liver disease, and hyperthyroidism, and patients who were pregnant were also excluded
Berber et al. (35)	From 2010 to 2021	PE	243	243/59	NR	Acute toxic hepatitis hospitalized	Incomplete patient records
Maheshwari et al. (36)	From January 2012 to September 2015	PE	NR	NR	NR	Patients underwent therapeutic plasma exchange	Patients who developed sepsis or multiorgan dysfunction prior to the PE and patients with the category IV indication, were excluded from the study
Meijers et al. (37)	From August 2008 and June 2009	MARS	27	27/10	NR	Acute-on-chronic liver failure	Sever hypocalcemia (Ca_2_^+^ < 0.9 mmol/L); acidosis (pH < 7.25) due to any cause
Falkensteiner et al. (38)	From 2012 to 2017	MARS	75	75/49	8 (6.8-8.0)	LF	Age < 18 years
Faybik et al. (39)	From January 2007 to May 2009	MARS	77	77/20	20 (18-22)	LF	Age < 18 years
Yonekawa et al. (40)	NR	CRRT + PE	39	39/9	NR	LF	NR

LF, liver failure; RRT, renal replacement therapy; CRRT, continuous renal replacement therapy; MARS, molecular adsorbent recirculation system; PE, plasma exchange; NR, not reported; RCA, regional citrate anticoagulation; Caion, ionized calcium; LMWH, low molecular weight heparin; ICG-PDR, indocyanine green plasma disappearance rate; ACLF, acute-on-chronic liver failure; HBV-ACLF, hepatitis B virus (HBV) related ACLF.

There were 7 studies that reported filter coagulation during ECOS, in which 6 were related to RRT, and 1 was related to MARS (Figure 2B). The overall incidence of filter coagulation was 4.4% [95%CI (1.6, 8.3)], and the incidence during RRT was 4.0% [95%CI (1.3, 7.9)]. There was no statistical difference in the incidence of filter coagulation between RRT group and MARS group, while significant heterogeneity was observed (Chi2 = 28.90, df = 6, *P* < 0.01, I2 = 79%).

#### 3.4.4. Safety

Safety was assessed according to the incidence of citrate accumulation and bleeding during ECOS, changes of hepatic and renal function indicators, and changes of electrolytes. Likewise, we focused on RRT, PE, and MARS.

#### 3.4.5. Citrate accumulation

**FIGURE 3:**
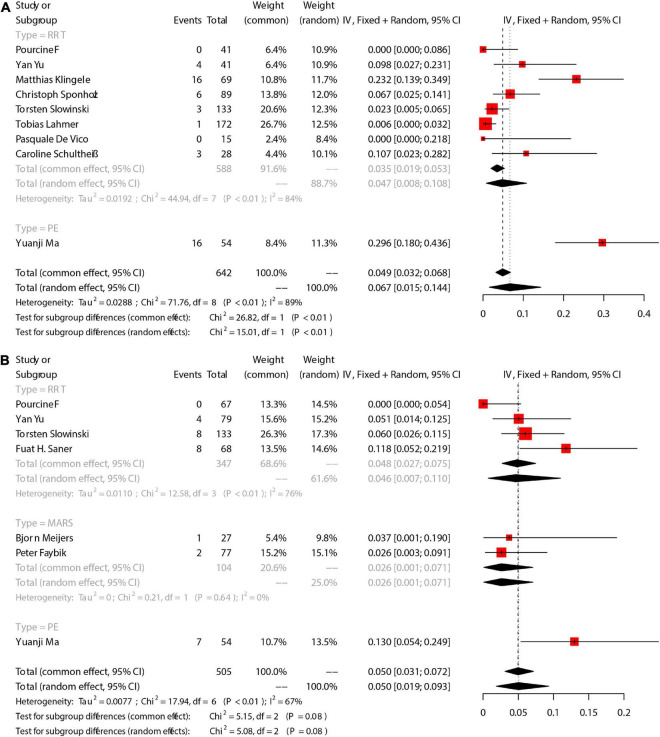
The pooled rates (95% CI) of Citrate accumulation **(A)** and bleeding **(B)**. RRT, renal replacement therapy; MARS, molecular adsorbent recirculation system; PE, plasma exchange; CI, confidence interval.

There were 9 studies that reported the number of patients with citrate accumulation, in which 8 was RRT and the other 1 was PE. Citrate accumulation was confirmed according to an Ca_tot_/Ca_ion_ ≥ 2.5. The incidence of citrate accumulation was 6.7% [95%CI (1.5, 14.4), Figure 3A]. Grouping analysis was performed for RRT-involved studies, and the pooled results showed that the incidence of citrate accumulation was 4.7% [95%CI (0.8, 10.8)]. There was only 1 study that reported citrate accumulation during PE, and the incidence was 29.6% [95%CI (18.0, 43.6)]. There was statistical difference in the incidence of citrate accumulation between RRT group and PE group (Chi2 = 15.01, df = 1, *p* < 0.01), and significant heterogeneity existed between the groups (Chi2 = 71.76, df = 8, *P* < 0.01, I2 = 89%).

**FIGURE 4:**
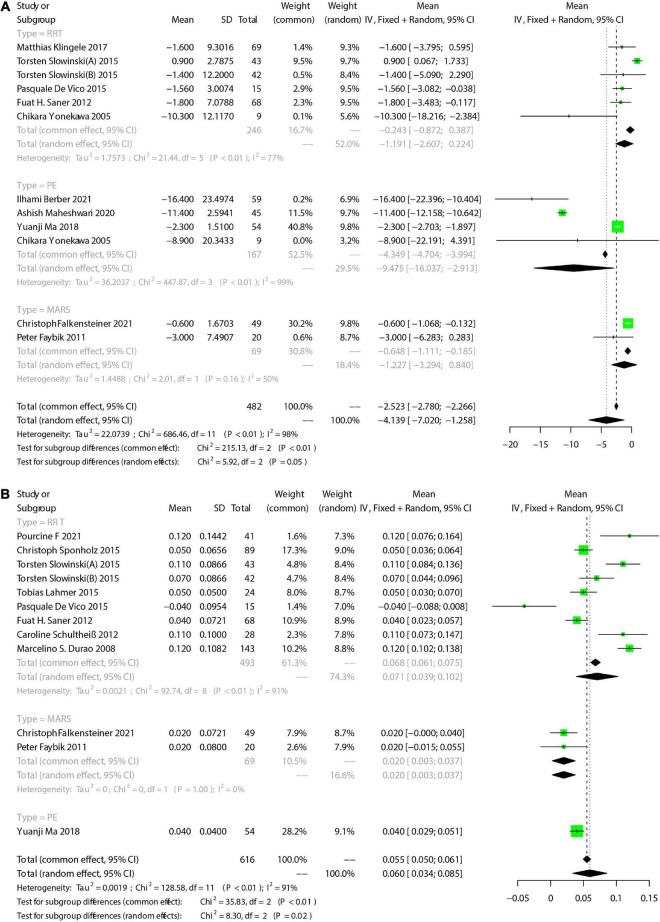
The pooled rates (95% CI) of TBIL **(A)** and pH **(B)**. RRT, renal replacement therapy; MARS, molecular adsorbent recirculation system; PE, plasma exchange; CI, confidence interval; SD, standard deviation; TBIL, total bilirubin; pH, potential of hydrogen.

On the other hand, 6 studies provided specific changes of Ca_tot_/Ca_ion_ ratio, and we compared the Ca_tot_/Ca_ion_ ratio before ECOS with that after ECOS to further explore the citrate accumulation during RCA. Generally, Ca_tot_/Ca_ion_ ratio slightly increased during ECOS [Mean = 0.141, 95%CI (0.053, 0.229)] (Figure 4). Pooled analysis for 3 RRT-involved studies showed an increased Ca_tot_/Ca_ion_ ratio [Mean = 0.136, 95%CI (0.017, 0.255)], and that for 2 PE-involved studies also showed an increased Ca_tot_/Ca_ion_ ratio [Mean = 0.190, 95%CI (−0.112, 0.492)].

#### 3.4.6. Bleeding

There were 7 studies that reported the number of patients with bleeding. The incidence of bleeding was 5.0% [95%CI (1.9, 9.3)] (Figure 3B), and 4 RRT-involved studies showed an incidence of 4.6% [95%CI (0.7, 11)]. Additionally, 2 MARS-involved studies showed an incidence of 2.6% [95%CI (0.1, 7.1)].

#### 3.4.7. Liver function

We focused on total bilirubin (TBIL), alanine transaminase (ALT), and aspartate aminotransferase (AST). There were 11 studies reported TBIL. Meta-analysis showed that the TBIL decreased after ECOS [Mean = −4.139, 95%CI (−7.020, −1.258), Figure 4A]. Subgroup analysis showed no significant change of TBIL in 5 RRT-involved studies [Mean = −1.197, 95%CI (−2.607, 0.224)]. In addition, 2 MARS-involved studies also showed no significant change of TBIL [Mean = −1.227, 95%CI (−3.249, 0.840)]. Pooled 4 PE-involved studies showed a significant decrease of TBIL [Mean = −9.475, 95%CI (−16.037, −2.913)]. We also assessed changes of ALT and AST before and after ECOS, and the results are shown in Table 4. Forest plots are provided in Supplementary Appendix 1, 2.

**TABLE 4:** Data integration results of different characteristics.

Outcomes	Type	Number of articles	Number of patients	Estimator	*I* ^2^
Ca_ton_/Ca_ion_	RRT	3	168	0.136 (0.017-0.255)	85
	PE	2	63	0.190 (0.112-0.492)	90
	MARS	1	20	0.130 (0.017-0.277)	NR
	Overall	6	251	0.141 (0.053-0.229)	81
ALT	RRT	2	153	−158.139 (−496.000-179.721)	76
	PE	3	158	−58.164 (−63.825-−52.502)	0
	MARS	1	20	−174.000 (−2,398.462-2,050.462)	NR
	Overall	6	331	−58.189 (−63.844-−52.534)	40
AST	RRT	2	153	−419.222 (−1,217.130-378.686)	79
	PE	3	158	−82.961 (−127.781-−38.140)	66
	MARS	1	20	−180.000 (−1,335.565-975.565)	NR
	Overall	6	331	−86.038 (−129.676-−42.400)	62
Creatinine	RRT	5	400	−0.746 (−1.096-−0.396)	90
	MARS	2	69	−0.294 (−0.517-−0.071)	0
	PE	2	99	−0.032 (−0.084-0.019)	0
	Overall	9	569	−0.515 (−0.793-−0.238)	95
BUN	RRT	5	400	−48.043 (−68.331-−27.756)	97
	MARS	1	49	0.000 (−23.809-23.809)	0
	Overall	6	449	−41.714 (−62.925-−20.502)	96
Platelet	RRT	2	153	−4.886 (−15.243-5.470)	0
	MARS	3	79	−4.354 (−15.935−7.244)	0
	PE	2	99	−26.153 (−39.229-−13.078)	0
	Overall	7	331	−10.214 (−16.865-−3.563)	33
APTT	RRT	2	153	−5.613 (−11.668-0.441)	75
	MARS	2	59	0.410 (−2.910-3.730)	0
	Overall	4	212	−2.919 (−7.659-1.820)	73
INR	RRT	2	153	−0.198 (−0.393-−0.003)	79
	PE	3	158	−0.673 (−1.347-0.001)	90
	Overall	5	311	−0.393 (−0.734-−0.053)	87
HCO_3_^–^	RRT	8	493	3.670 (2.024-5.315)	93
	MARS	1	20	1.000 (−0.753-2.753)	NR
	PE	1	54	2.000 (0.911-3.089)	NR
	Overall	10	567	3.284 (1.848-4.719)	93
BE	RRT	6	437	4.752 (3.174-6.329)	88
	MARS	1	20	2.400 (−0.098-4.898)	NR
	Overall	7	457	4.501 (3.026-5.976)	87
PCO_2_	RRT	5	193	0.553 (−1.867-2.974)	66
	MARS	1	20	1.000 (−4.259-6.259)	NR
	PE	1	54	−1.000 (−2.600-0.600)	NR
	Overall	7	267	0.356 (−1.492-2.205)	63
Acidosis	RRT	4	247	0.220 (0.088-0.388)	84
Alkalosis	RRT	5	427	0.065 (0.006-0.169)	89
Lactate	RRT	5	307	−0.540 (−1.169-0.088)	74
	MARS	1	49	0.200 (−0.139-0.539)	NR
	PE	1	54	0.200 (0.040-0.360)	NR
	Overall	7	410	0.286 (−0.793-0.221)	88
Na	RRT	6	261	0.424 (−1.247-2.095)	81
	MARS	1	20	1.000 (−1.319-3.319)	NR
	PE	1	54	4.200 (2.773-5.627)	NR
	Overall	8	335	0.926 (−0.615-2.468)	84
K	RRT	4	192	−0.155 (−0.249-0.060)	39
	MARS	1	20	−0.100 (−0.275-0.075)	NR
	PE	1	54	0.400 (0.225-0.575)	NR
	Overall	6	266	−0.077 (−0.261-0.107)	84
Hypocalcemia	RRT	4	4,544	0.053 (0.023-0.092)	86
Hypercalcemia	RRT	4	1,112	0.086 (0.002-0.256)	96
Sepsis	RRT	6	386	0.379 (0.238-0.530)	88
	PE	1	54	0.296 (0.180-0.436)	NR
	Overall	7	440	0.367 (0.246-0.497)	87
Los_ICU	RRT	6	237	16.397 (6.789-26.004)	96

RRT, renal replacement therapy; CRRT, continuous renal replacement therapy; MARS, molecular adsorbent recirculation system; PE, plasma exchange; Caion, ionized calcium; Caton, total calcium; ALT, alanine transaminase; AST, aspartate aminotransferase; BUN, blood urea nitrogen; APTT, activated partial thromboplastin time; INR, international normalized ratio; BE, base excess; ICU, intensive care unit; los_ICU, length of ICU stay; NR, not reported.

#### 3.4.8. Renal function

Studies that assessed renal function were RRT-involved. We reviewed data of serum creatinine (SCr) and blood urea nitrogen (BUN), as shown in Table 4. Forest plots are presented in Supplementary Appendix 3, 4. RRT resulted in significantly decreased SCr and BUN.

#### 3.4.9. Coagulation function

Coagulation function-associated indicators were also changed during ECOS. We primarily assessed platelet (PLT), Activated partial thromboplastin time (APTT), and international normalized ratio (INR) (Table 4). There were 7 studies that reported PLT, and the results showed a decrease of PLT after ECOS [Mean = −10.214, 95%CI (−16.865, −3.563), Supplementary Appendix 5]. Subgroup analysis showed that the decrease of PLT was statistically significant only in PE group [Mean = −26.153, 95%CI (−39.229, −13.07)], while not in RRT and MARS group. There were 4 studies that reported the change of APTT, with no statistical significance [Mean = −2.919, 95%CI (−7.659, 1.820), Supplementary Appendix 6]. In addition, 5 studies reported the change of INR, and the pooled results are shown in Table 4.

#### 3.4.10. Acid-base and electrolyte status

The acid-base equilibrium fluctuated during ECOS. Meta-analysis showed that pH [Mean = 0.060, 95%CI (0.034, 0.05), Figure 4B], BE [Mean = 3.284, 95%CI (1.848, 4.719), Supplementary Appendix 7], BE [Mean = 4.501, 95%CI (3.026, 5.976), Supplementary Appendix 8], and SPCO_2_ [Mean = 0.356, 95%CI (−1.492, 2.205), Supplementary Appendix 9]. The incidence of metabolic acidosis during RCA-ECOS was 22% [95%CI (8.8, 38.8), Supplementary Appendix 10], and that of metabolic alkalosis was 6.5% [95%CI (0.6, 16.9), Supplementary Appendix 11]. Blood lactic acid [Mean = 0.286, 95%CI (-0.793, 0.221), Supplementary Appendix 12]. In addition, we pooled the changes of Na^2+^, K^+^, and the incidence of hypercalcemia and hypocalcemia, and the results are shown in Table 4 and Supplementary Appendix 13–16.

#### 3.4.11. Other results

We also assessed the development of sepsis during ECOS and ICU-duration. Meta-analysis showed that the incidence of sepsis during RCA-ECOS was 36.7 [95%CI (24.6, 49.7), Supplementary Appendix 17], and the mean ICU-duration was 16.397 [95%CI (6.789, 26.004), Supplementary Appendix 18].

### 3.5. Sensitivity analysis and publication bias

**FIGURE 5:**
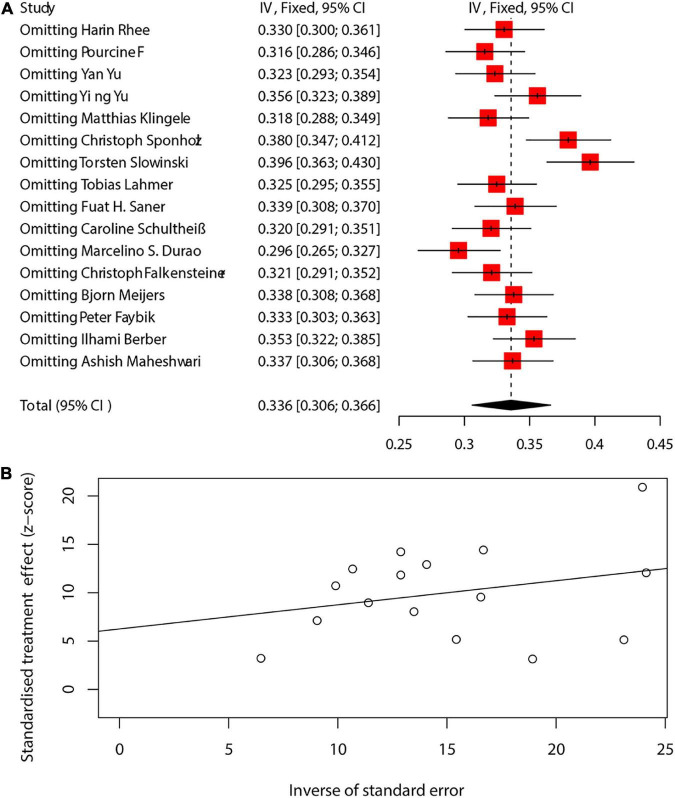
The sensitivity and publication bias of the main results of mortality **(A,B)**. CI, confidence interval.

We analyzed the sensitivity of the pooling effect of more than 10 articles. The results of sensitivity analysis are shown in Supplementary Appendix 19–22. There was no significant change in the pooled effect, indicating that the results were robust. Publication bias was evaluated using funnel plot and Egger’s test. We assessed the sensitivity and publication bias of the main results of mortality (Figure 5A). A relatively symmetric funnel plot indicated no significant publication bias, which was validated by Egger’s test (*p* = 0.1025) (Figure 5B). The power of the Egger’s test might be limited if less than 10 studies included, so that the difference between contingency and true asymmetry would not be distinguished. We presented the Egger’s test of more than 10 studies in the Supplementary Appendix 23–26. No potential publication bias was observed in the included studies. Not all tests are available for subset datasets with a small sample size.

## 4. Discussion

Liver failure is a clinical syndrome commonly seen in clinical practice. It is of rapid progression and high mortality (42). The primary treatment strategies include comprehensive physical treatment, artificial liver support, stem cell transplantation, and liver transplantation (42). Studies have shown that the incidence of AKI ranges from 40% to 85% in LF patients (22, 43), and ECOSs such as RRT, PE, and MARS are recommended, for patients with treatment failure or waiting for transplantation, to alleviate the capacity load, remove the solute of small molecules, correct the acid-base imbalance and electrolyte disturbance, so as to stabilize the internal environment. ECOS can create conditions for patients to recover or transition to liver transplantation, combined liver and kidney transplantation and other further treatment. There are multiple patterns of bleeding and coagulation disorders in patients with liver failure. Except calcium ion and coagulation factor VIII, other coagulation factors are synthesized in the liver. In patients with liver insufficiency, the synthesis of coagulation factors and anticoagulants decreased, and the coagulation system of patients with liver failure was rebalanced, but the balance was brittle balance, which was easily broken by hemostasis or anticoagulation therapy, causing bleeding or hypercoagulability (44, 45). Moreover, the contact of blood with biomaterial of filter during ECOS could cause bioincompatible reaction (46), including the activation of coagulation cascade and cell-mediated inflammatory response (47). Therefore, one major problem of ECOS is the anticoagulation in extracorporeal circulation. unfractionated heparin (UFH) and low molecular weight heparin (LMWH) remain the preferred coagulants in most trial centers. However, heparin would probably induce systemic anticoagulation so that aggravate LF-related coagulation disorder (48). In the past years, citrate has become an effective alternative to heparin for ECOS anticoagulation (49).

It presents a challenge for the ECOS anticoagulation in LF patients admitted to ICU. Here we have reviewed the results of 19 studies, in which 12 applied RCA during RRT, 3 during PE, 3 during MARS, and 1 during RRT-PE combination. Citrate is a vital intermediate substance for tricarboxylic acid cycle in mitochondria, and its final metabolites are carbon dioxide and water. Citrate usually exists in mitochondria-rich organs such as liver, kidney, and muscles due to its metabolism demanding oxygen (50). Exogenous citrate serves as a calcium ion chelating agent in anticoagulation, and calcium ion is an important cofactor in coagulation cascade. This makes RCA a highly effective anticoagulant in ECOS. Several studies have found that the use of citrate for anticoagulation in ICU-admitted patients could reduce bleeding-associated complications and prolong the filter lifespan, and might reduce mortality (51) When RCA is used in ECOS, calcium citrate complex can be partially removed by filter, and tricarboxylic acid cycle is mainly carried out in the liver after the rest of the calcium citrate complex enters the body. Therefore, the use of RCA in patients with LF is controversial (52). The reason might be the complexity of citrate. It requires specific citrate solution/alternative solution. One the other hand, LF patients might have increased risk for citrate accumulation and unpredictable metabolic complications (such as acid-base imbalance and electrolyte disturbance).

Liver failure is a heterogeneous disease state, and multiple liver support technologies have been developed to attenuate the accumulation of endogenous toxins so as to improve the outcome of LF (53). Currently, PE and MARS are the most widely applied treatments (54). Moreover, other organ dysfunction often occurs in LF patients, such as previously mentioned end-stage liver disease and liver transplantation. RRT would be necessary in that these complications could cause AKI (55). CRRT is applied in most cases in ICU. It is usually the most preferable RRT for patients with AKI (56). In 2003, a pharmacokinetic study, which involved a cohort of 16 patients with liver cirrhosis, aroused interest in the use of RCA for patients with liver dysfunction (52). RCA-RRT has been acclaimed in recent years (57), and has been recommended by KDIGO guideline for anticoagulation (14, 58, 59). According to the guideline released by European Association for the Study of the Liver (EASL), RRT should be performed as early as possible for patients with sustained hyperammonemia, hyponatremia, metabolic disturbance, and abnormal body temperature (60). Multiple studies have demonstrated the feasibility of RCA in LF patients receiving RRT (12, 27–29). Citrate-based RRT can reduce the risk of bleeding, complications, and extracorporeal coagulation in patients with LF-induced coagulation dysfunction (28), and closely monitoring during RCA-RRT can probably avoid citrate accumulation (12, 27).

MARS is an adjuvant treatment system for liver diseases, mainly by removing endogenous and exogenous toxins to block the vicious circle, improve the balance of internal environment, reduce the continued destruction of hepatocytes, and promote the regeneration of hepatocytes and the functional recovery of critical hepatocytes (39, 61). MARS can not only reduce albumin and non-albumin binding toxins (62), but improve hemodynamics (63) and decrease bilirubin level in LF patients (64). Recent studies have shown that RCA could be safely applied in patients receiving MARS (38, 40). An observational study on 20 LF patients indicate that RCA would be safe, and no episodes of coagulation or bleeding observed during MARS (40). Another prospective study has revealed that RCA-MARS would be safe and feasible under close metabolic monitoring in LF patients, and would be more effective than non-RCA-MARS (38). As for survival, a study by Gerth et al. has shown that MARS could reduce the 28-day mortality of LF patients (61), which is supported by some other studies (39). However, there are some studies that hold different view on the survival benefit of MRAS (65). Two studies indicate MARS brings no benefits to the survival of LF patients, and a study report a higher 28-day mortality in patients receiving MARS over 4 times, compared with those receiving MARS less than 4 times (66).

Plasma exchange is also a commonly used artificial liver support treatment, which uses a plasma separator to separate and discard patient’s plasma that contains pathological components, and supplement 2,000-3,000 ml of fresh frozen plasma that contains essential substances such as protein and coagulation factors (67). This process filters out plenty of bilirubin, endotoxin, and virus, and rapidly improves the internal environment so as to alleviate liver injury and improve the regeneration environment of hepatocytes (22, 68). Studies have shown that PE could reduce the risk of bleeding in ALF patients via correcting the coagulation function (69). PE can also improve cerebral blood and oxygen support (70). In recent years, there are not only animal studies that have validated the efficacy and safety of citrate-based PE (71), but clinical reports regarding the use of citrate in PE. Betz et al. applied Fresenius Ci-Ca, (72) and provide intravenous injection of calcium as needed under the mode of fixed citrate and calcium ratio, which provide a feasible approach for the use of PE in patients with high risk of bleeding. The main complication of PE is hypocalcemia. Fresh frozen plasma contains a large amount of citric acid preparation, which increases the risk of hypocalcemia and citrate accumulation (73). Slow intravenous administration (15-30 min) of 10% calcium chloride injection or continuous intravenous infusion of calcium gluconate after PE initiation can prevent citrate toxicity (74).

In this study, we have analyzed the results of 19 studies from 4 databases to assess the efficacy and safety of citrate as anticoagulant in ECOS. Firstly, citrate anticoagulation can provide adequate anticoagulation for blood during cardiopulmonary bypass, thereby minimizing coagulation disorders associated with contact activation while maintaining systemic coagulation (75). The liver contributes essentially to exogenous citric acid metabolism, and the clearance of endogenous citric acid is decreased in LF patients (52). As a result, metabolic acidosis and increased anion gaps may occur. According to previous studies, a serum Catot/Caion ratio greater than or equal to 2.5 may be the critical threshold for potential citrate accumulation (76). Secondly, although the use of ECOS is relatively safe, a considerable number of studies have reported the incidence of bleeding-associated complications during the treatment, ranging from 9% to 40% (77). A study of citrate anticoagulation in RRT reported no bleeding complications and no blood transfusion within 24 h after RRT (28). In addition, the effect of ECOS on renal function is also evident. RRT can improve renal function. At the same time, the MARS device is combined with routine hemodialysis for RRT. Therefore, creatinine and urea nitrogen levels were reduced during MARS treatment. Studies on changes in biochemical markers also reported at least a decrease in creatinine levels during the use of MARS (78). However, RRT is more effective in reducing SCr and BUN, compared with the 8-h course recommended by MARS, which might be attributed to the longer treatment duration. In current studies, MARS usually operates in CVVHD mode, and the effectiveness of renal replacement may be lower than that of CVVHD mode.

This is the first systematic review and meta-analysis conducted to assess the efficacy and safety of citrate anticoagulation in ECOSs, including RRT, MARS and PE. However, some limitations exist. There are differences in the basic characteristics of patients, such as disease severity, gender, age, regional population, and treatment regimen, leading to significant clinical heterogeneity among the studies. There are also a variety of models to be selected even in RRT. We failed to provide specific treatment, but unified as one study. In addition, the study-design are not unified. The inherent limitations of non-randomized studies in Meta-analysis may also affect our results. More prospective RCTs are needed to further validate our findings. The evaluation criteria for results may vary depending on the type of treatment used, combination therapy, the degree of liver failure, and the duration of ECOS treatment, especially in different countries. We tried to use random-effect model and subgroup analysis method to solve these problems. Meta-analysis is also limited by the quality of included studies. Because it is in a relatively new period of exploration and research, the potential publication bias could not be ignored. Considering the above limitations, the results of our meta-analysis should be interpreted prudently.

## 5. Conclusion

Citrate-based anticoagulation would be safe and effective in ECOS treatment for LF patients. Close monitoring and timely adjustment are needed during the process to avoid potential complications. Due to the quantitative and qualitative limitations in this study, more prospective clinical trials of high quality are needed to further validate our findings so as to provide more robust evidence for the anticoagulation strategy in ECOS for LF patients. Moreover, due to the interactions between organs, multi-organ support therapy can provide joint support for different organ failure, and the integrated multi-organ support platform is the future development direction, which makes it necessary to select the most preferable anticoagulation strategy.

## Data availability statement

The original contributions presented in this study are included in the article/supplementary material, further inquiries can be directed to the corresponding authors.

## Author contributions

BP and JLu wrote the main manuscript and fully participated in all analyses. AL and JLi contributed to the study concept and design. HG and JLu participated in literature search, data extraction, and quality assessment. All authors read and approved the final manuscript.
