# Supplementary figures and images for: Regional citrate anticoagulation for replacement therapy in patients with liver failure: A systematic review and meta-analysis
Source: Front Nutr. 2023 Feb 16;10:1031796. doi: 10.3389/fnut.2023.1031796 (PMC9977825; doi:10.3389/fnut.2023.1031796)

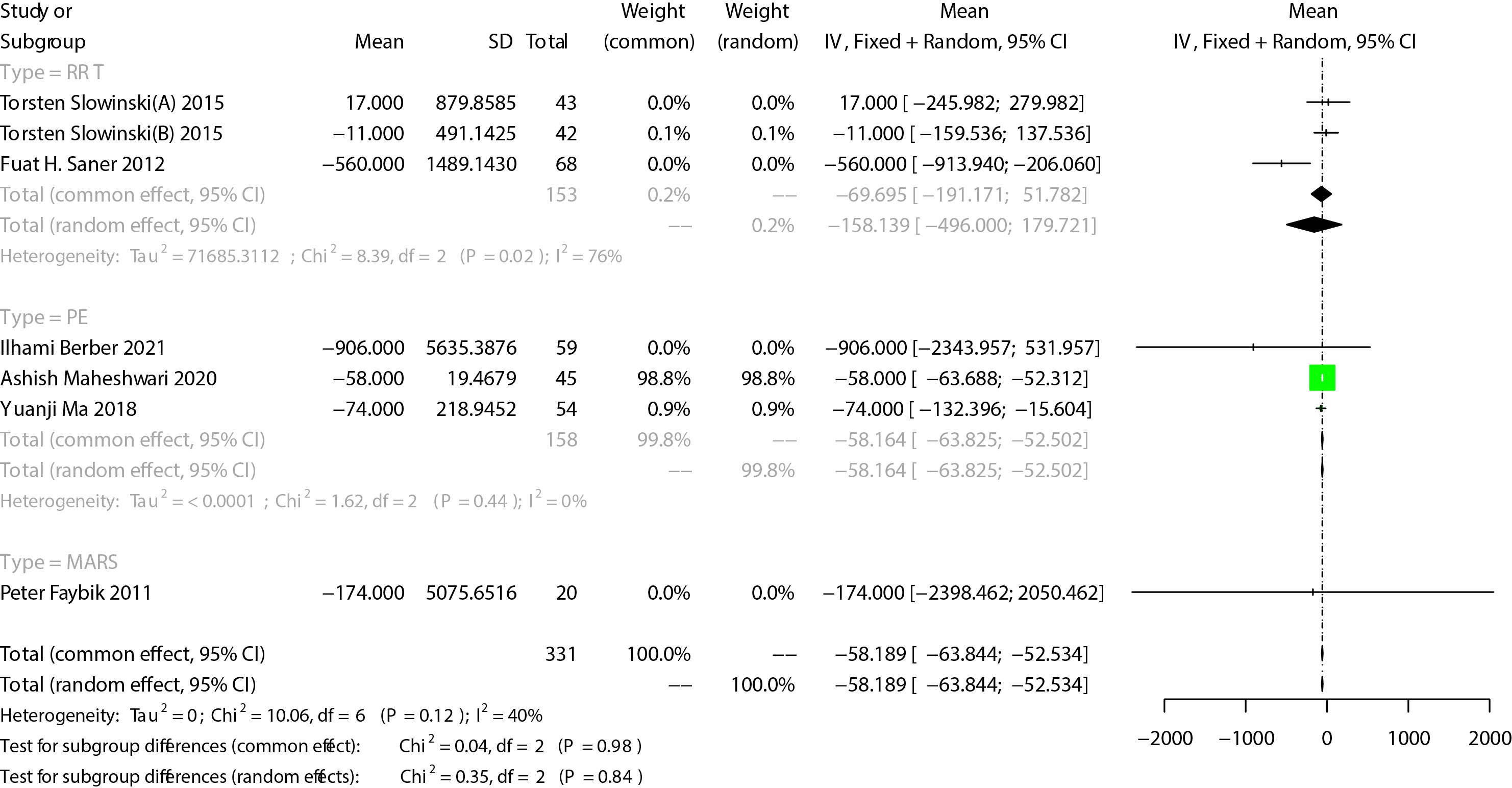

Supplement: Supplementary file 1 [file Presentation_1.zip › 文章补充图/Appendix 1.jpg]

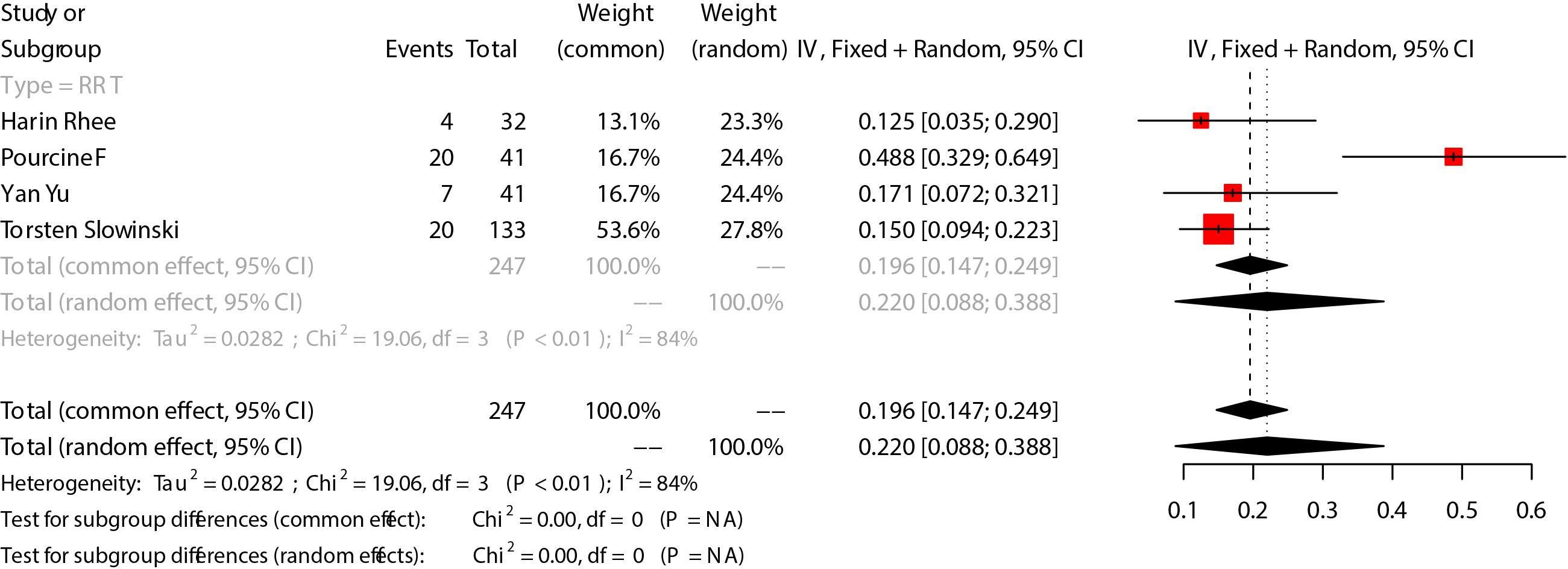

Supplement: Supplementary file 1 [file Presentation_1.zip › 文章补充图/Appendix 10.tif]

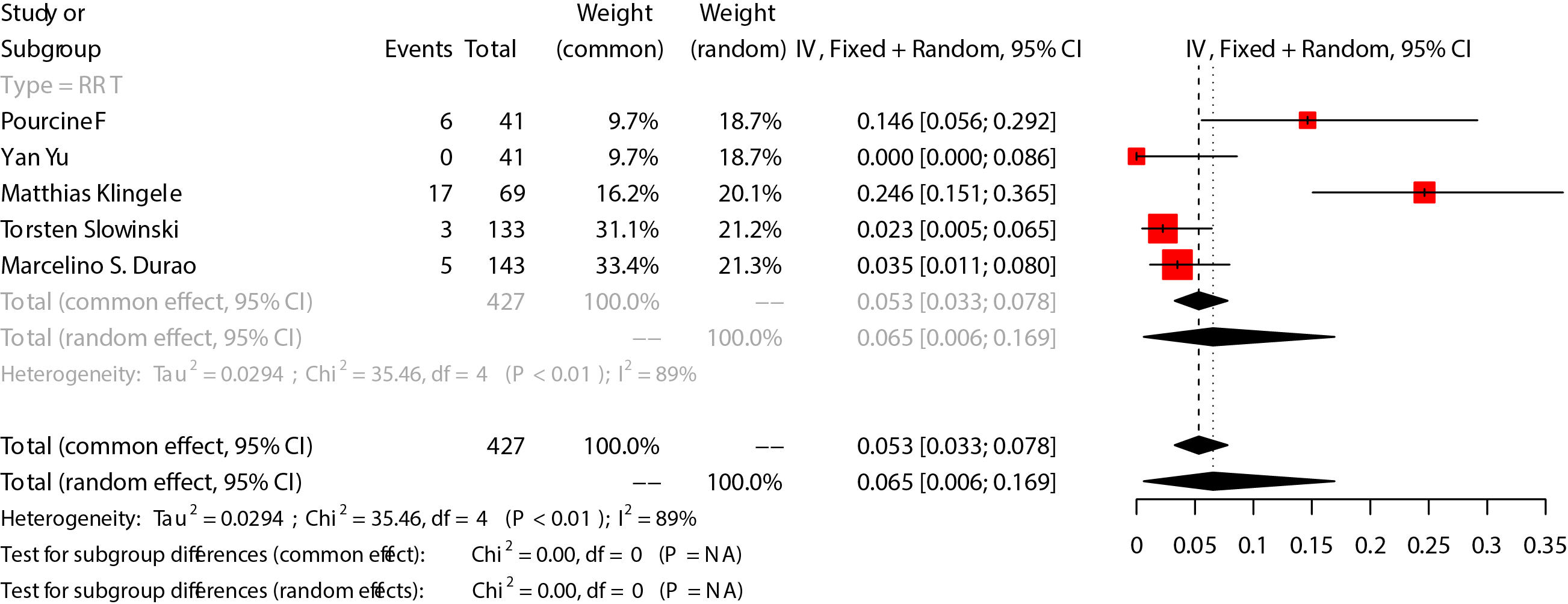

Supplement: Supplementary file 1 [file Presentation_1.zip › 文章补充图/Appendix 11.tif]

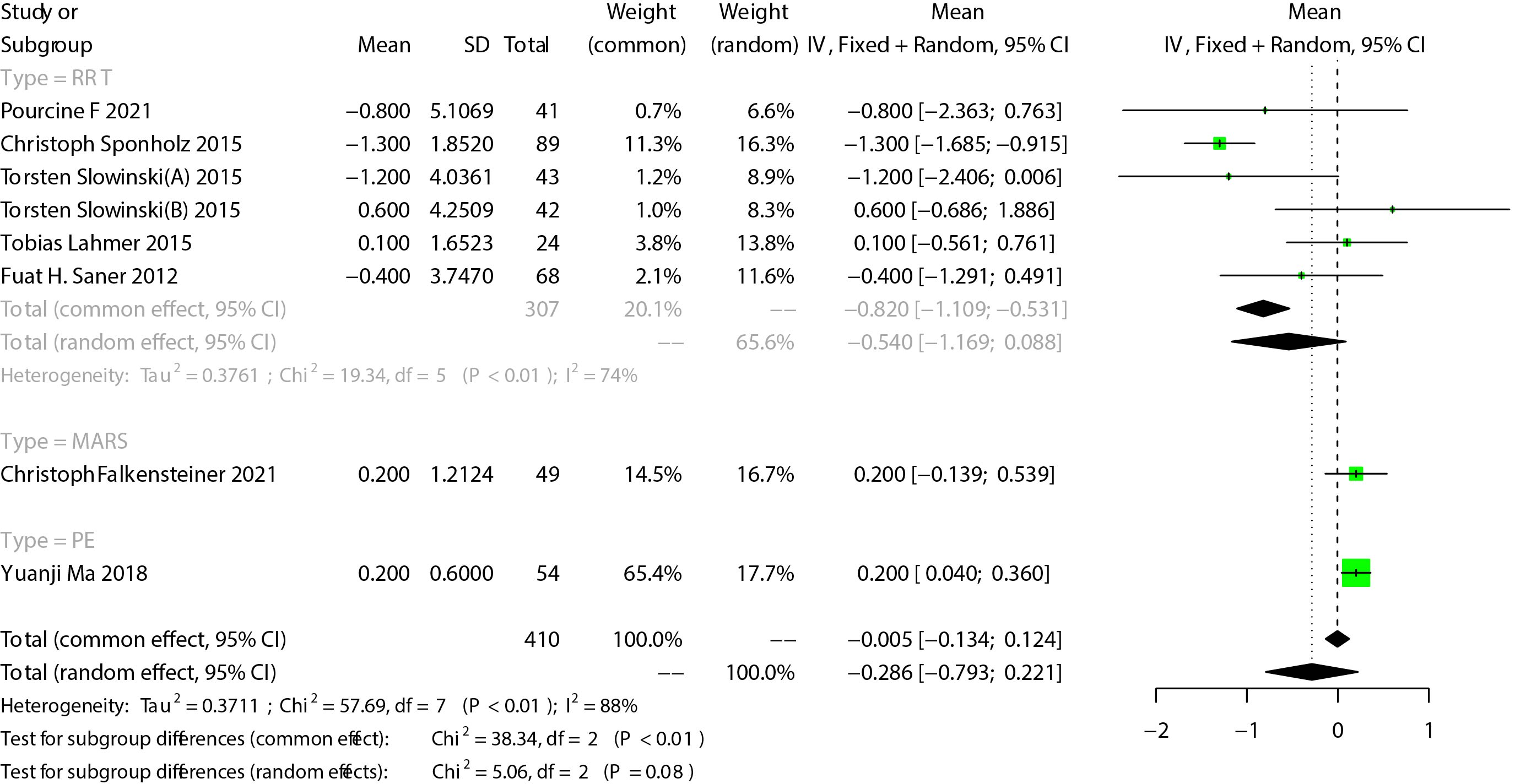

Supplement: Supplementary file 1 [file Presentation_1.zip › 文章补充图/Appendix 12.jpg]

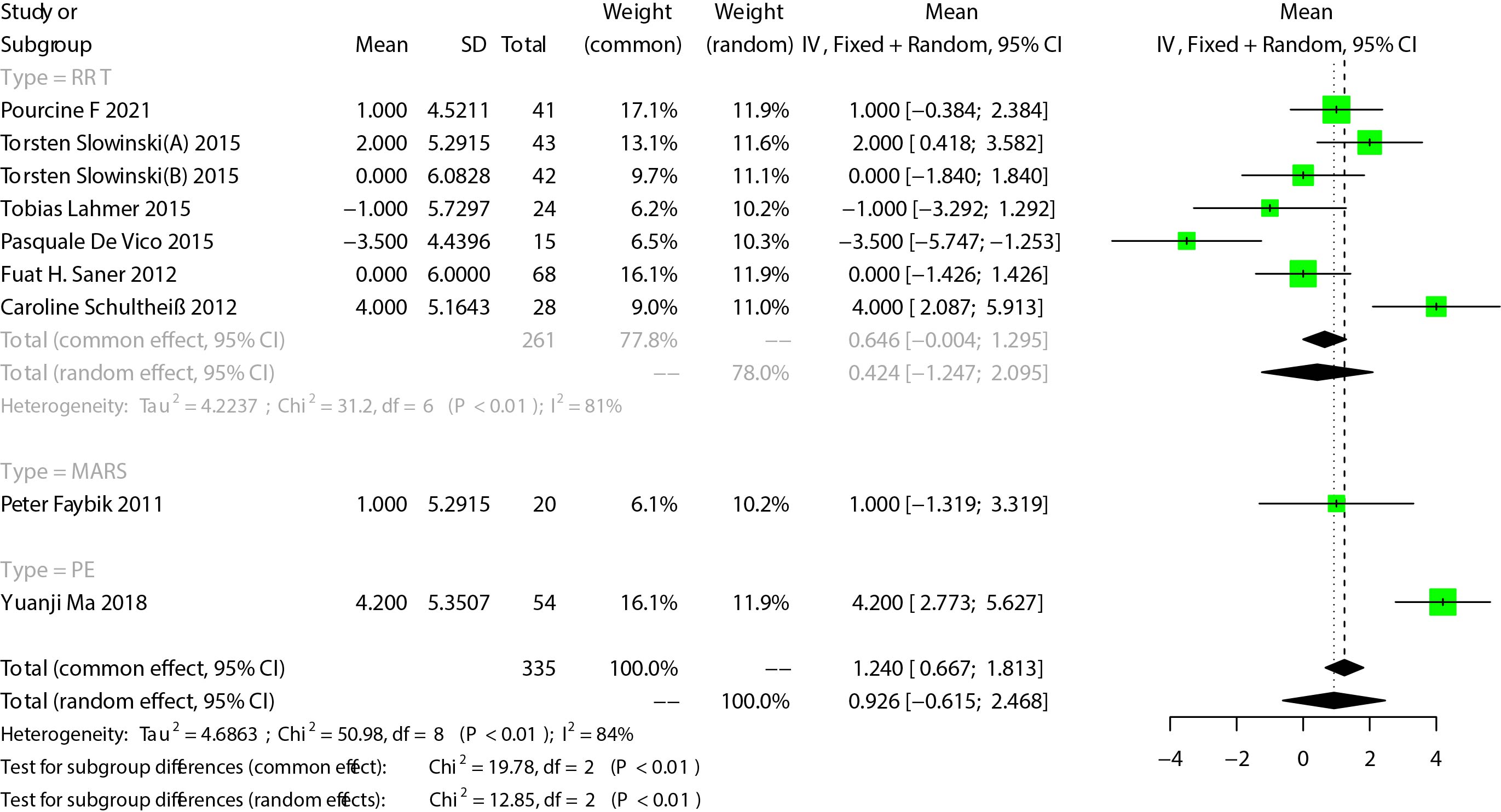

Supplement: Supplementary file 1 [file Presentation_1.zip › 文章补充图/Appendix 13.jpg]

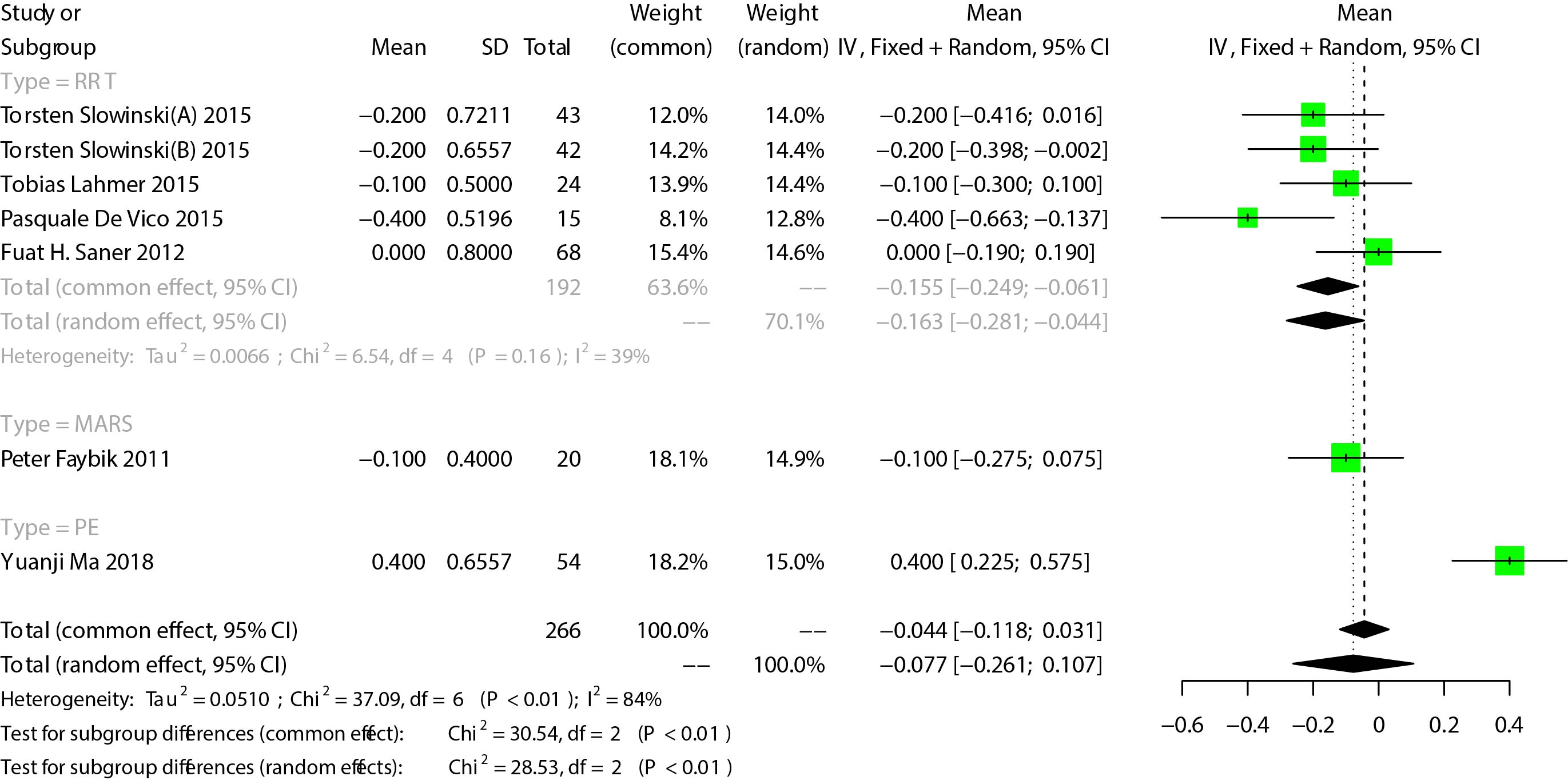

Supplement: Supplementary file 1 [file Presentation_1.zip › 文章补充图/Appendix 14.jpg]

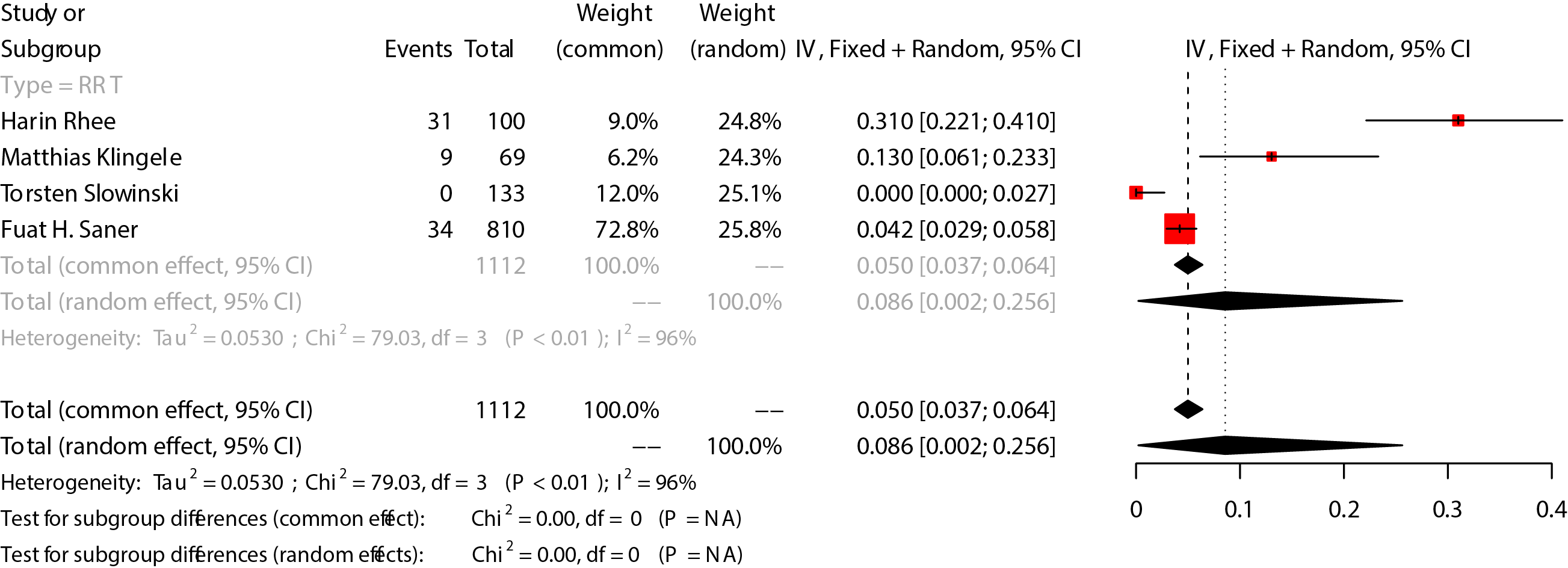

Supplement: Supplementary file 1 [file Presentation_1.zip › 文章补充图/Appendix 15.tif]

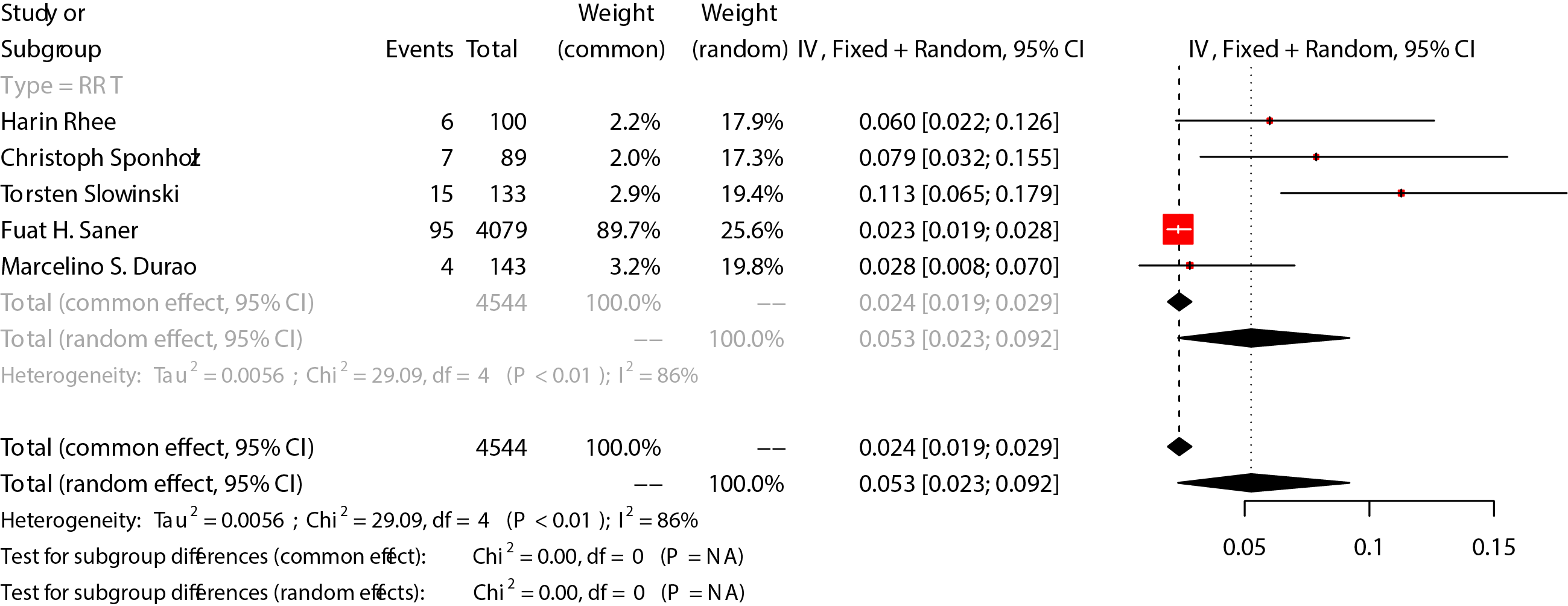

Supplement: Supplementary file 1 [file Presentation_1.zip › 文章补充图/Appendix 16.tif]

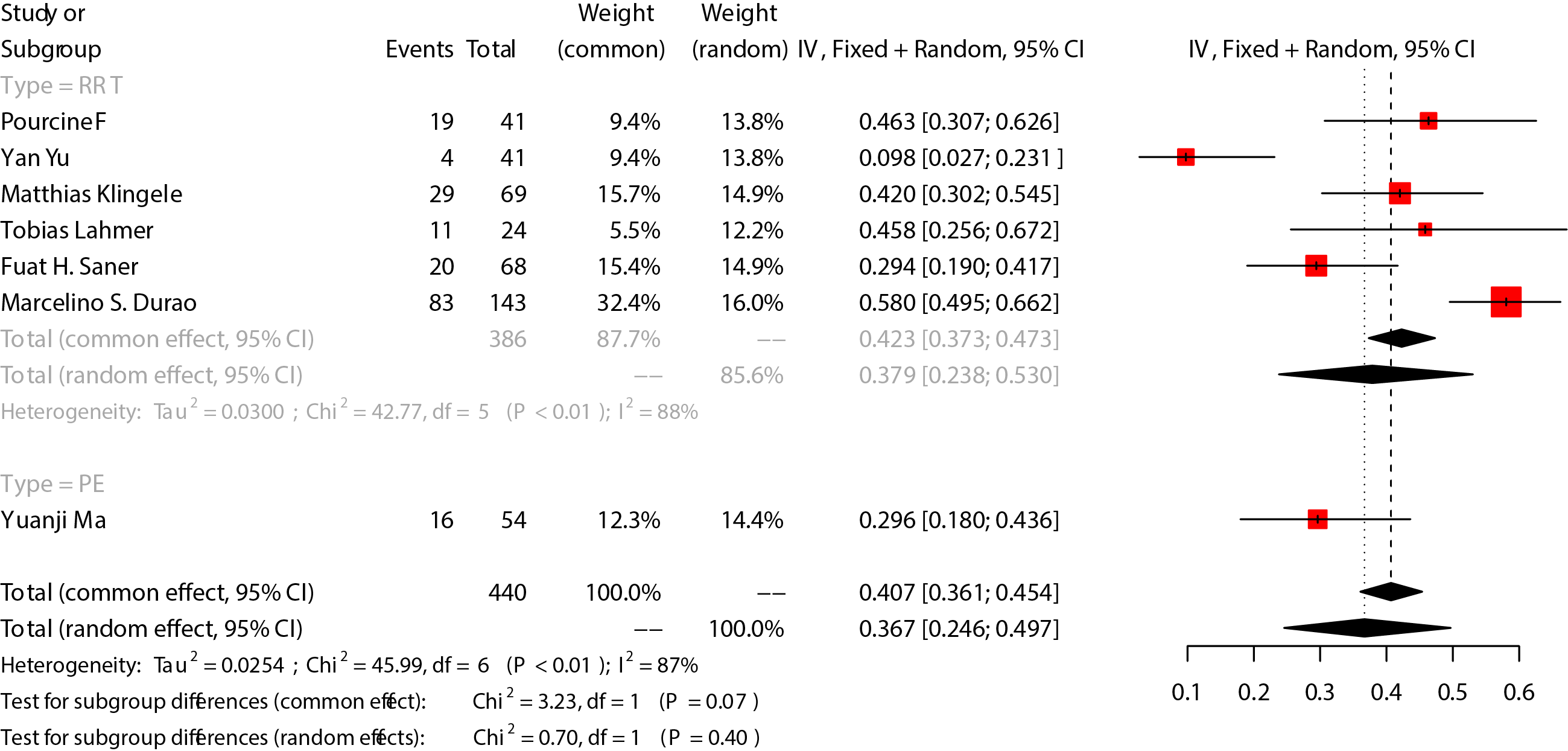

Supplement: Supplementary file 1 [file Presentation_1.zip › 文章补充图/Appendix 17.tif]

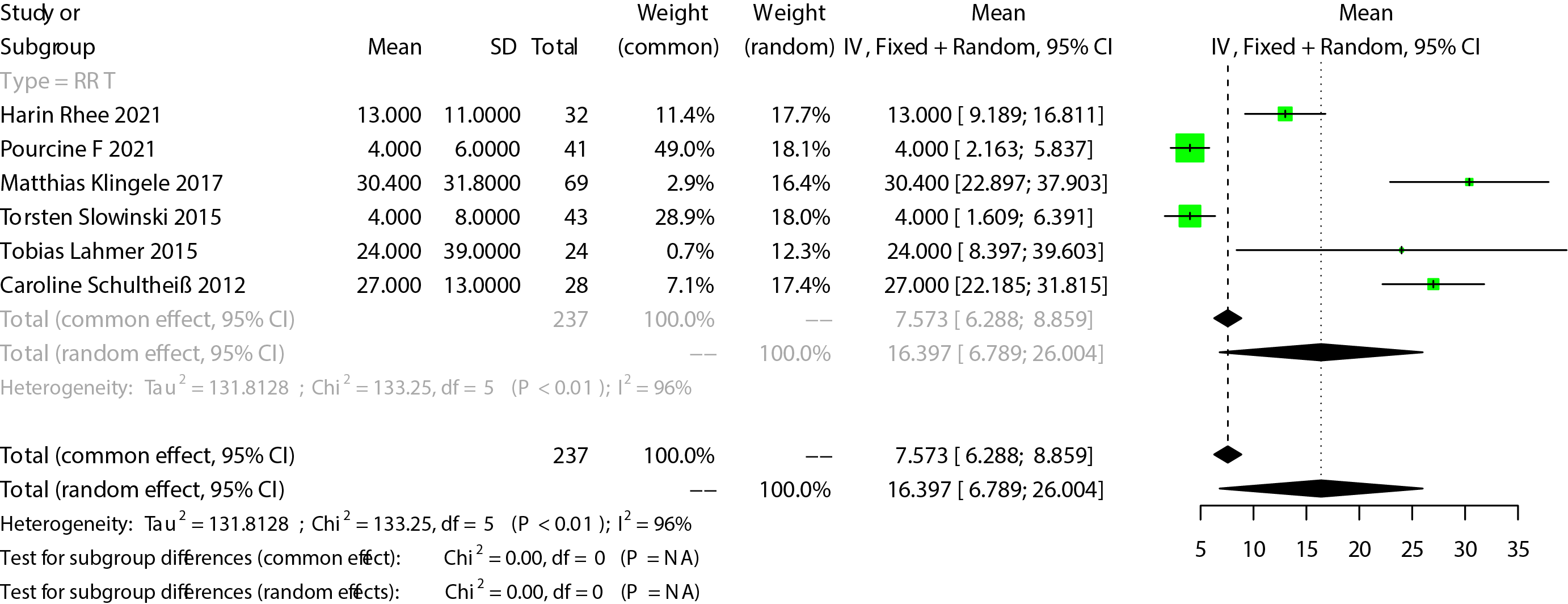

Supplement: Supplementary file 1 [file Presentation_1.zip › 文章补充图/Appendix 18.tif]

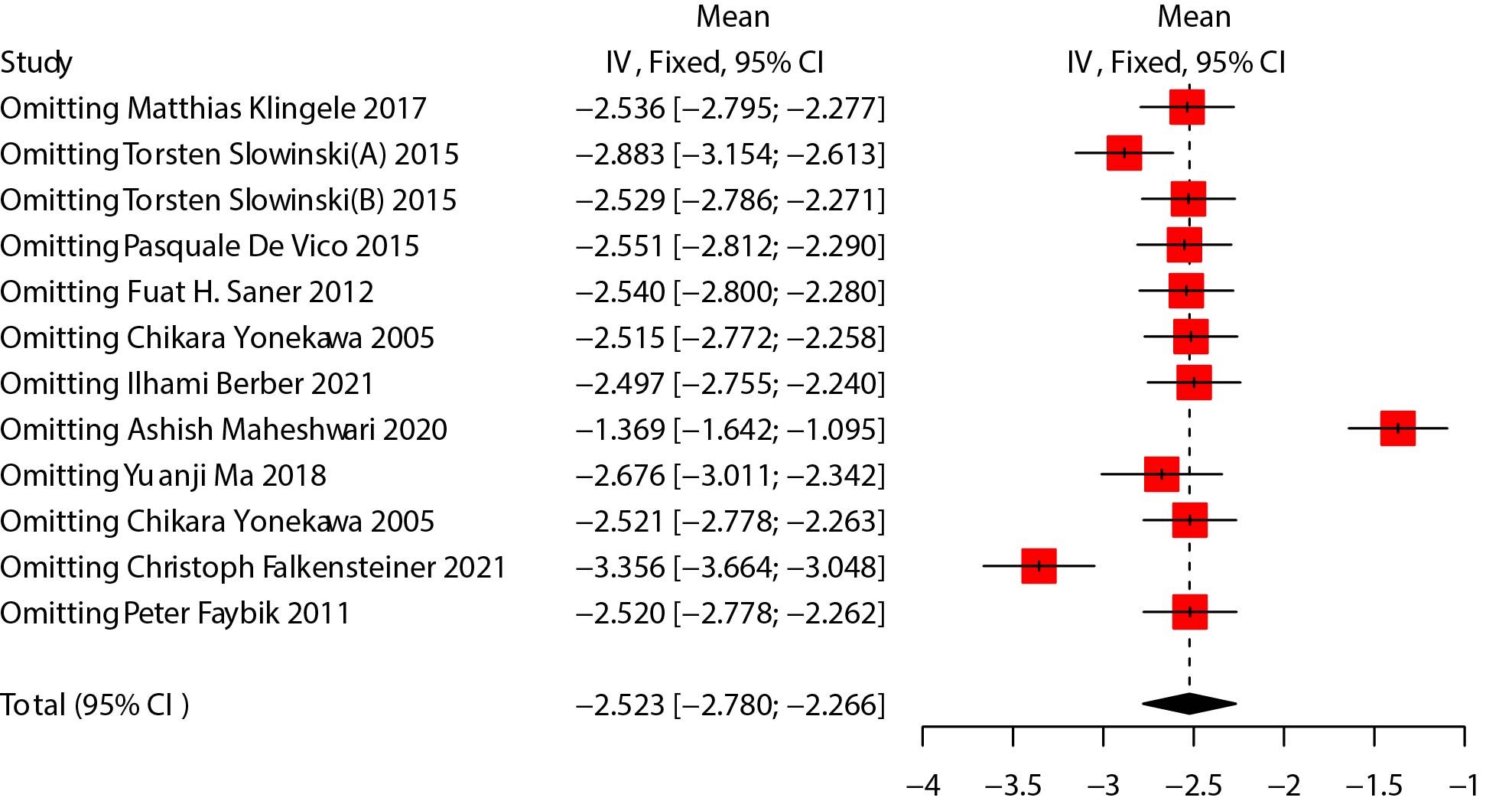

Supplement: Supplementary file 1 [file Presentation_1.zip › 文章补充图/Appendix 19.jpg]

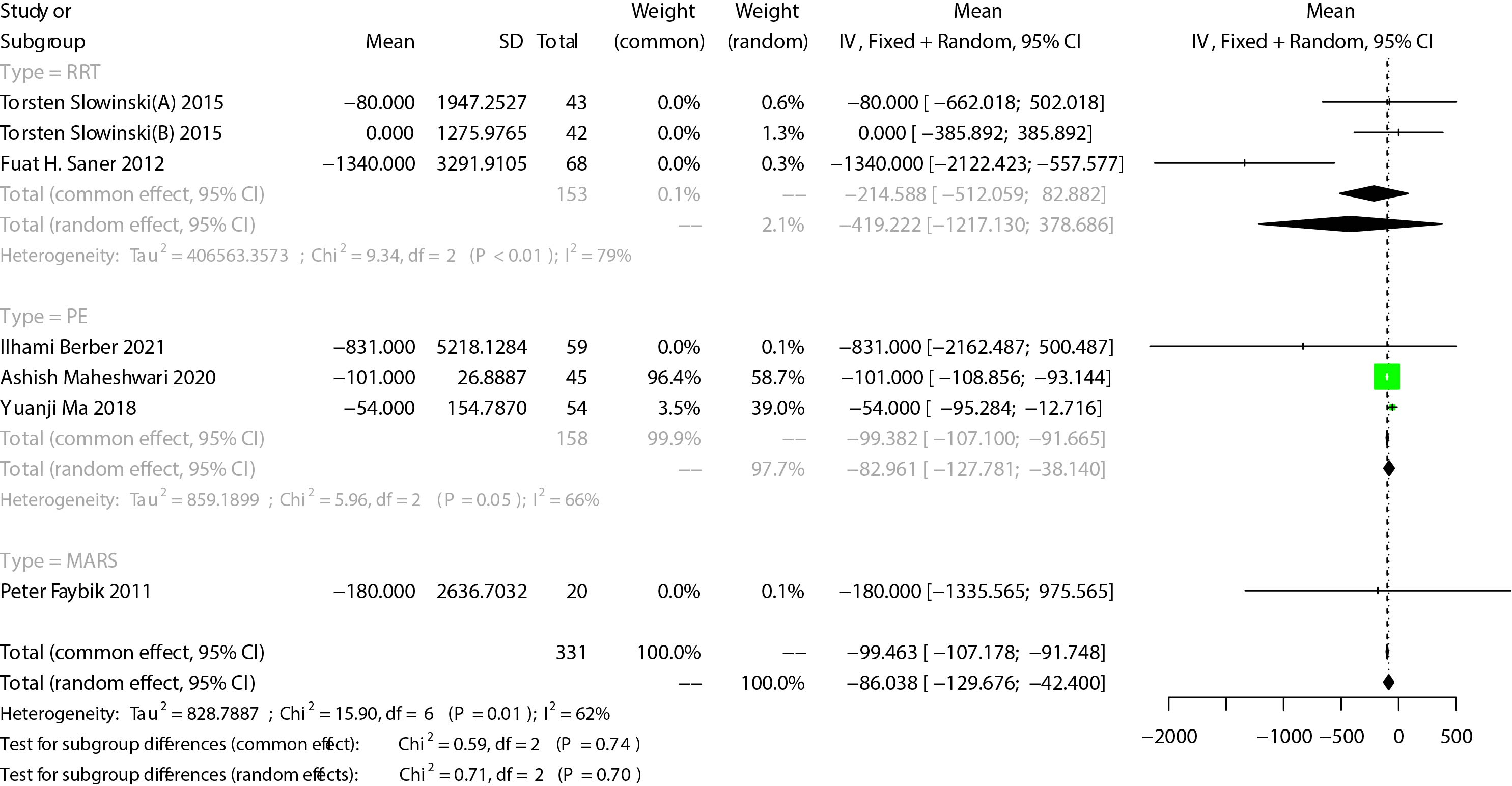

Supplement: Supplementary file 1 [file Presentation_1.zip › 文章补充图/Appendix 2.jpg]

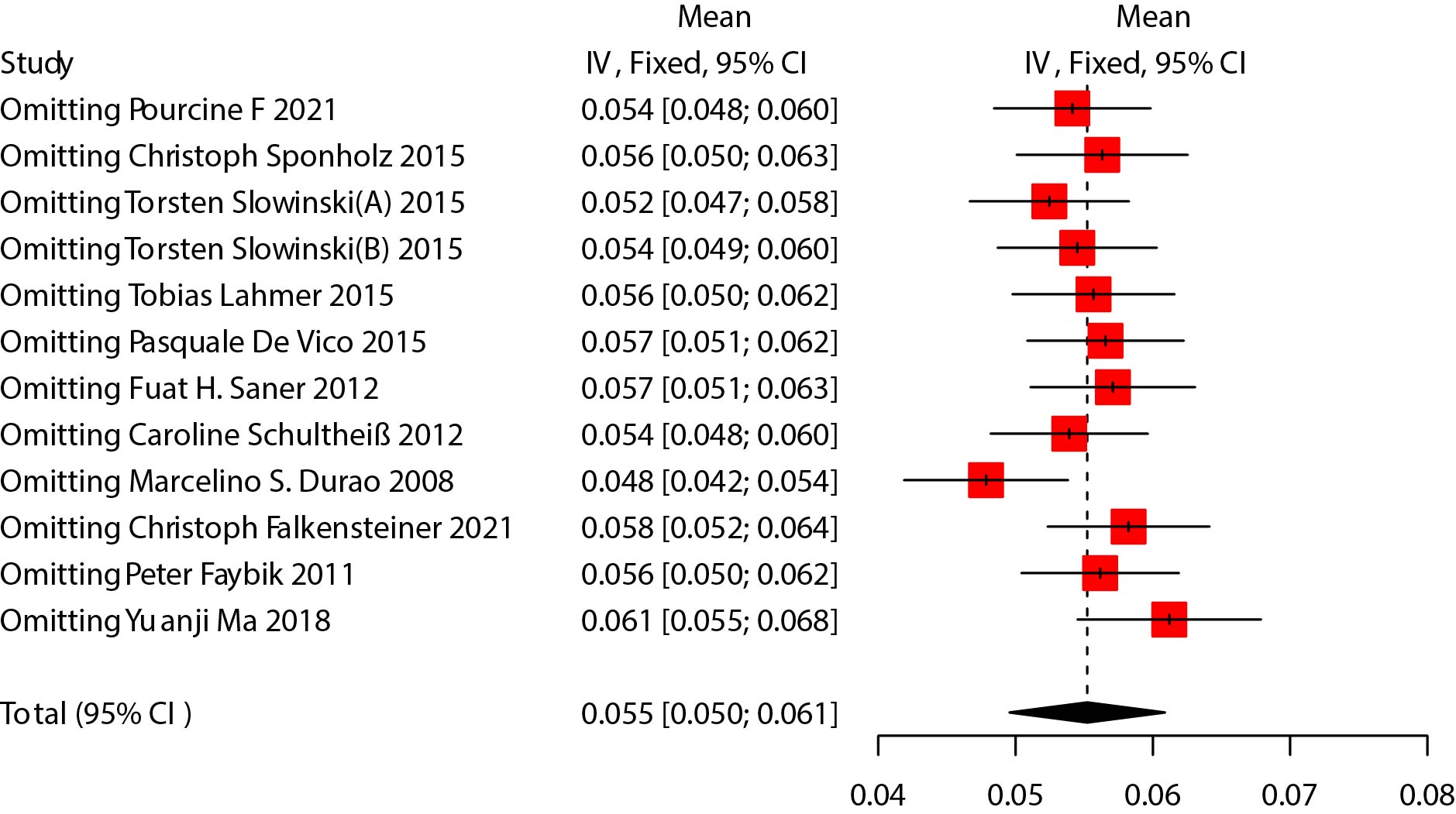

Supplement: Supplementary file 1 [file Presentation_1.zip › 文章补充图/Appendix 20.jpg]

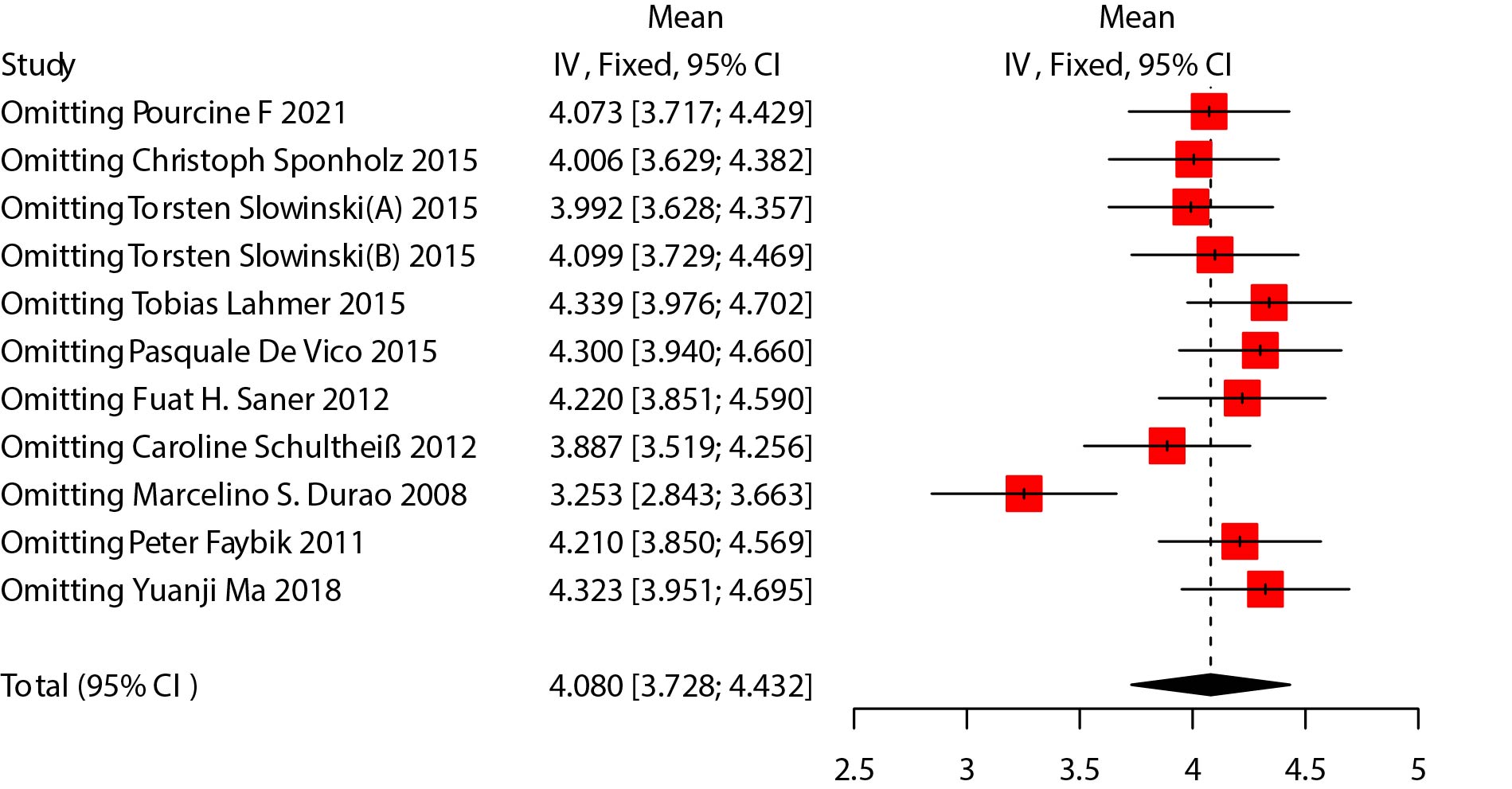

Supplement: Supplementary file 1 [file Presentation_1.zip › 文章补充图/Appendix 21.jpg]

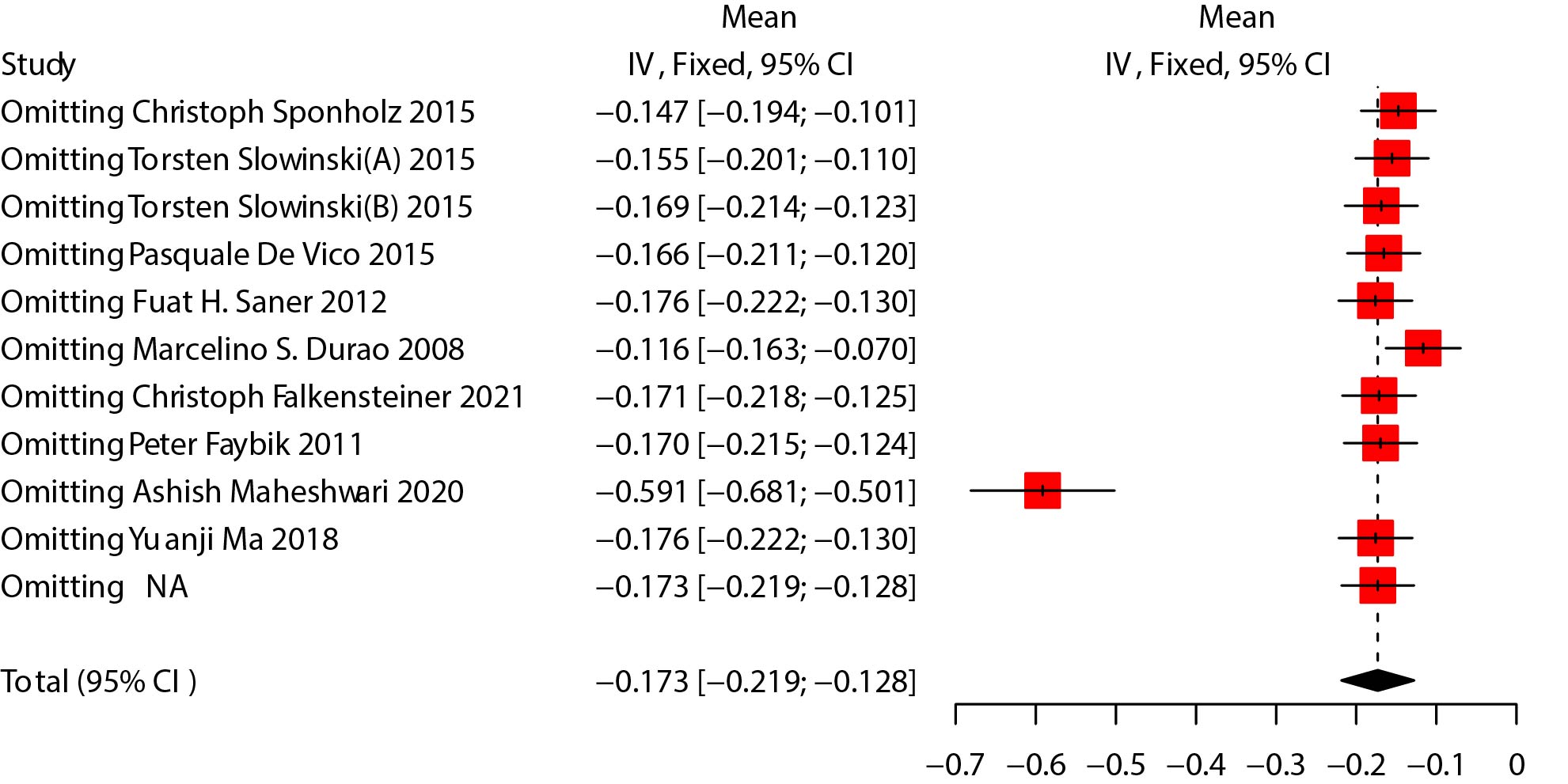

Supplement: Supplementary file 1 [file Presentation_1.zip › 文章补充图/Appendix 22.jpg]

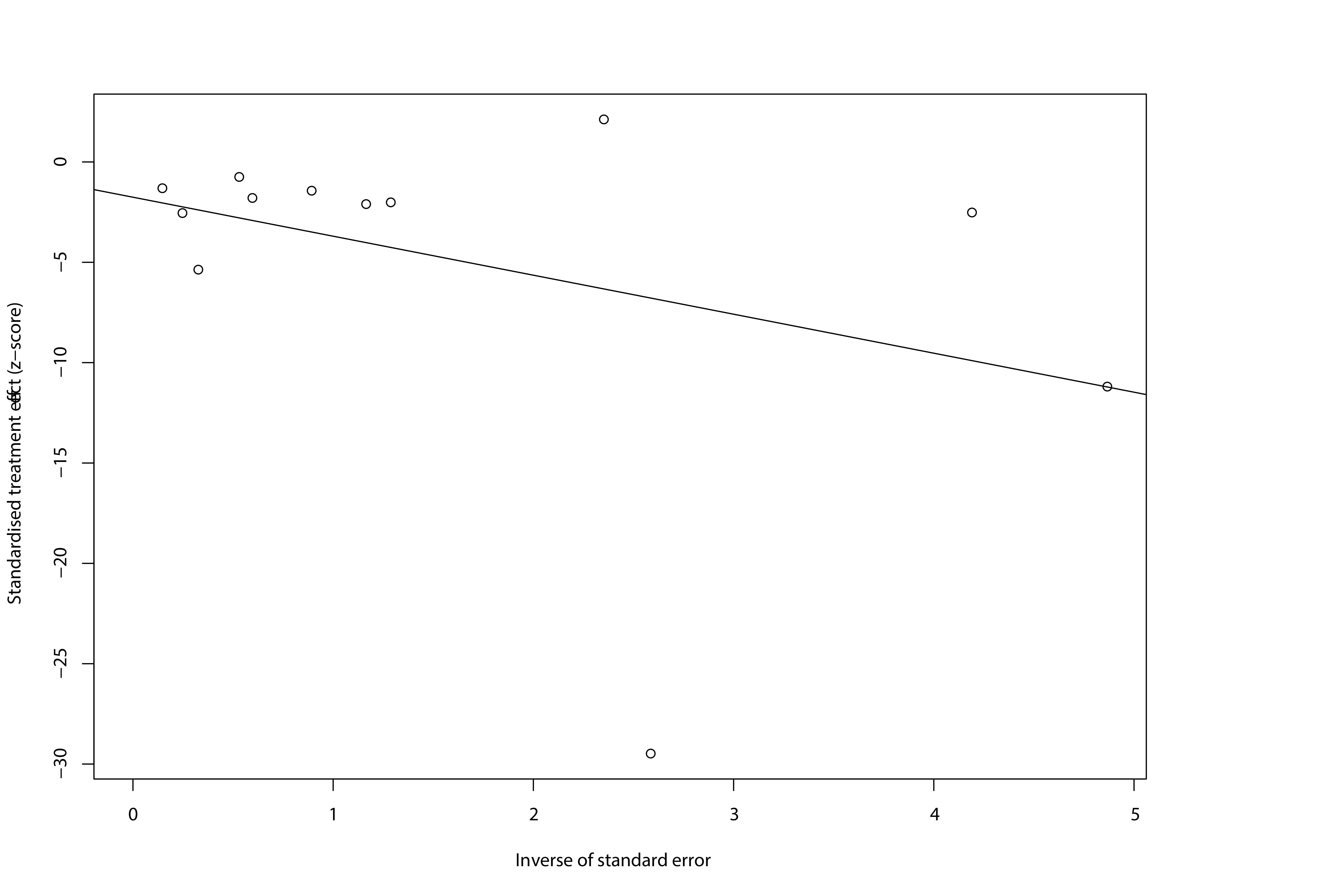

Supplement: Supplementary file 1 [file Presentation_1.zip › 文章补充图/Appendix 23.jpg]

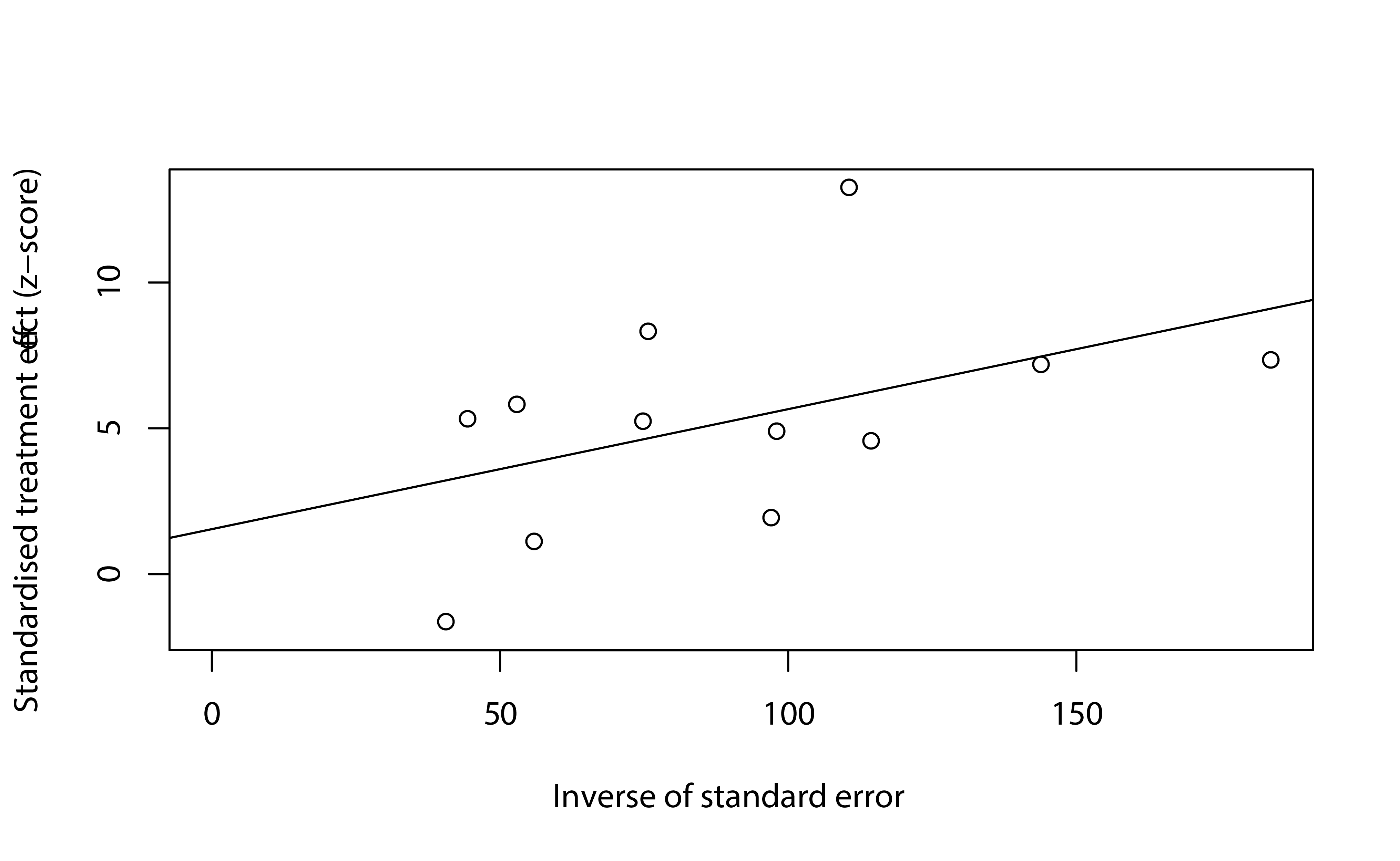

Supplement: Supplementary file 1 [file Presentation_1.zip › 文章补充图/Appendix 24.tif]

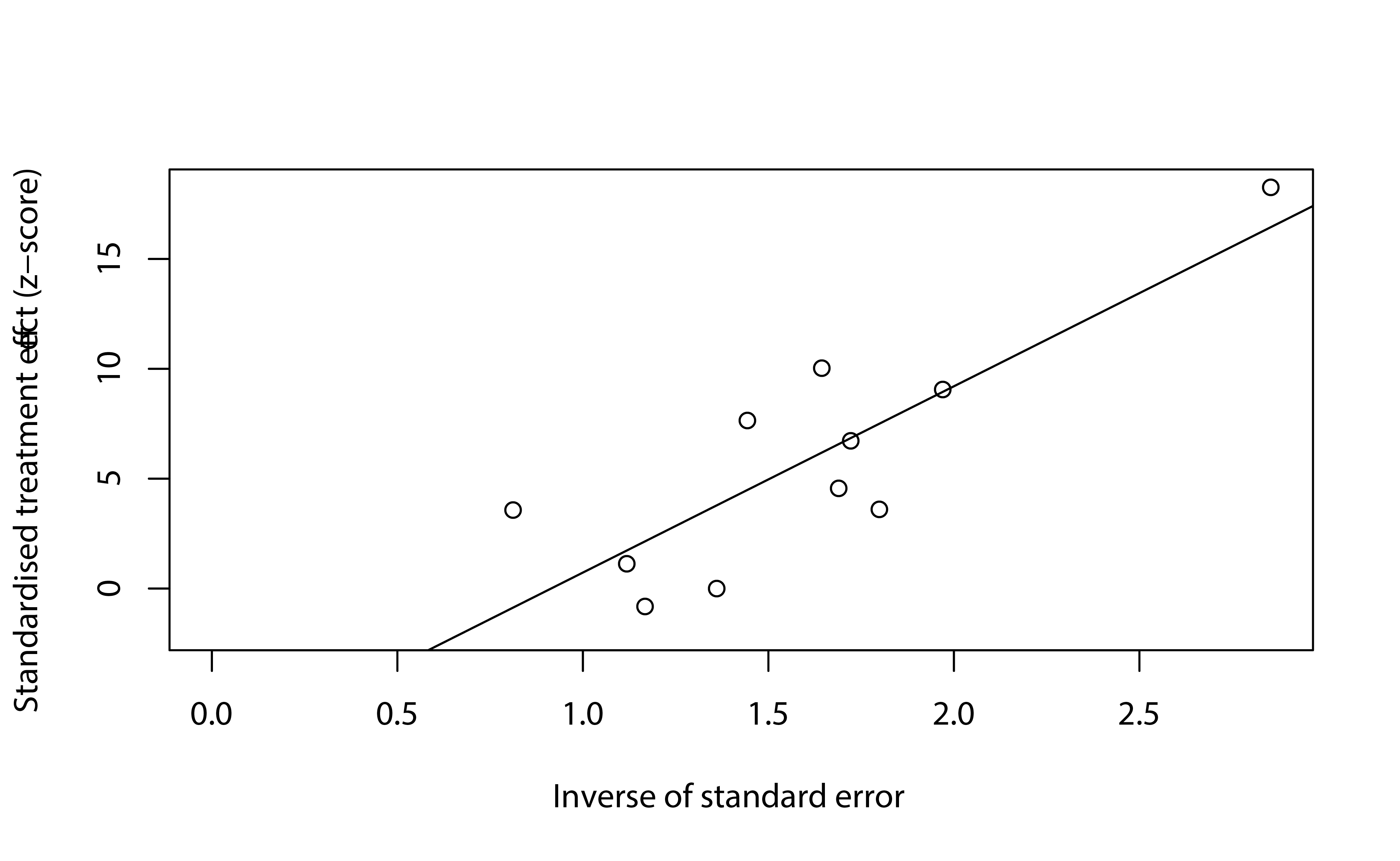

Supplement: Supplementary file 1 [file Presentation_1.zip › 文章补充图/Appendix 25.tif]

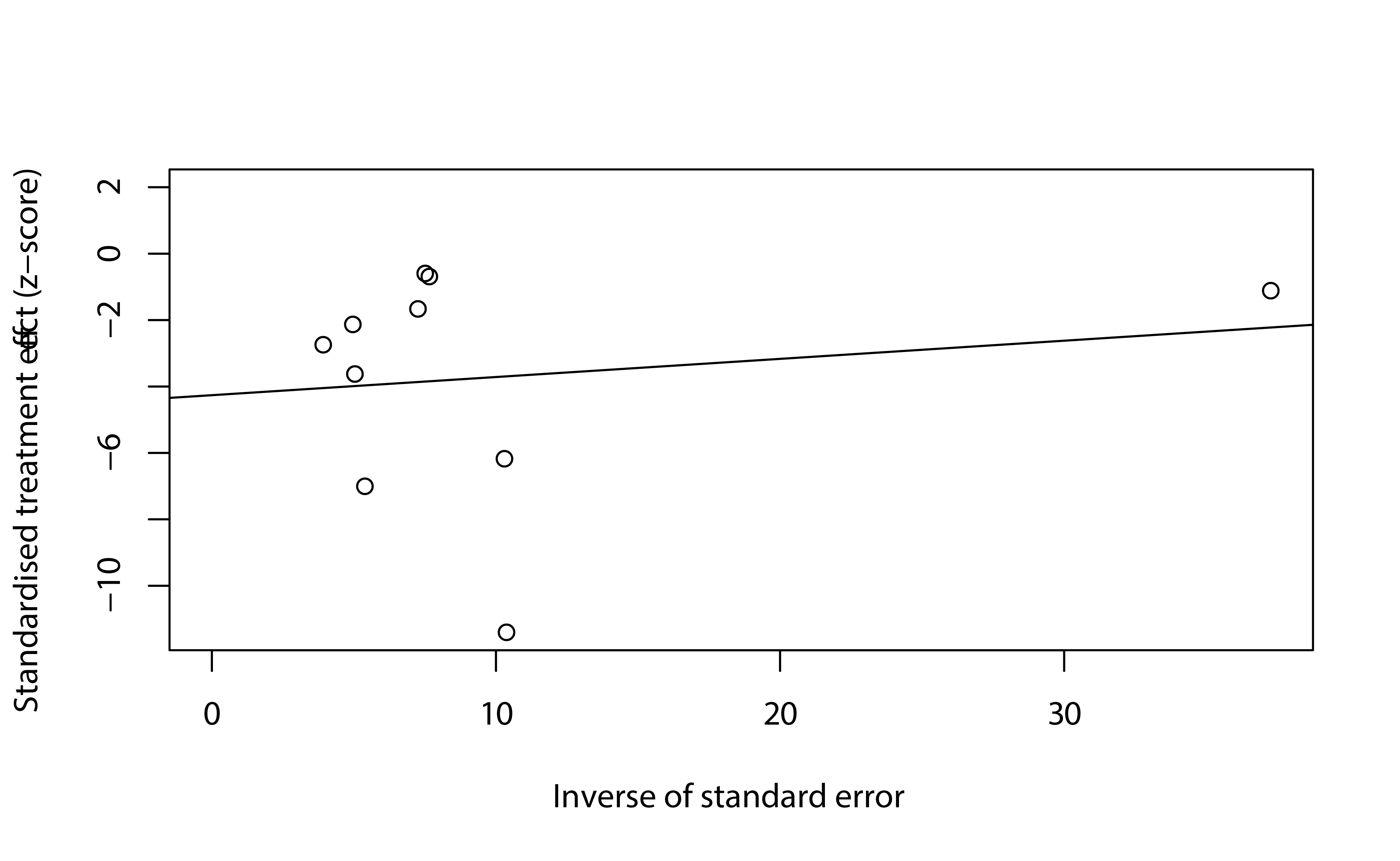

Supplement: Supplementary file 1 [file Presentation_1.zip › 文章补充图/Appendix 26.tif]

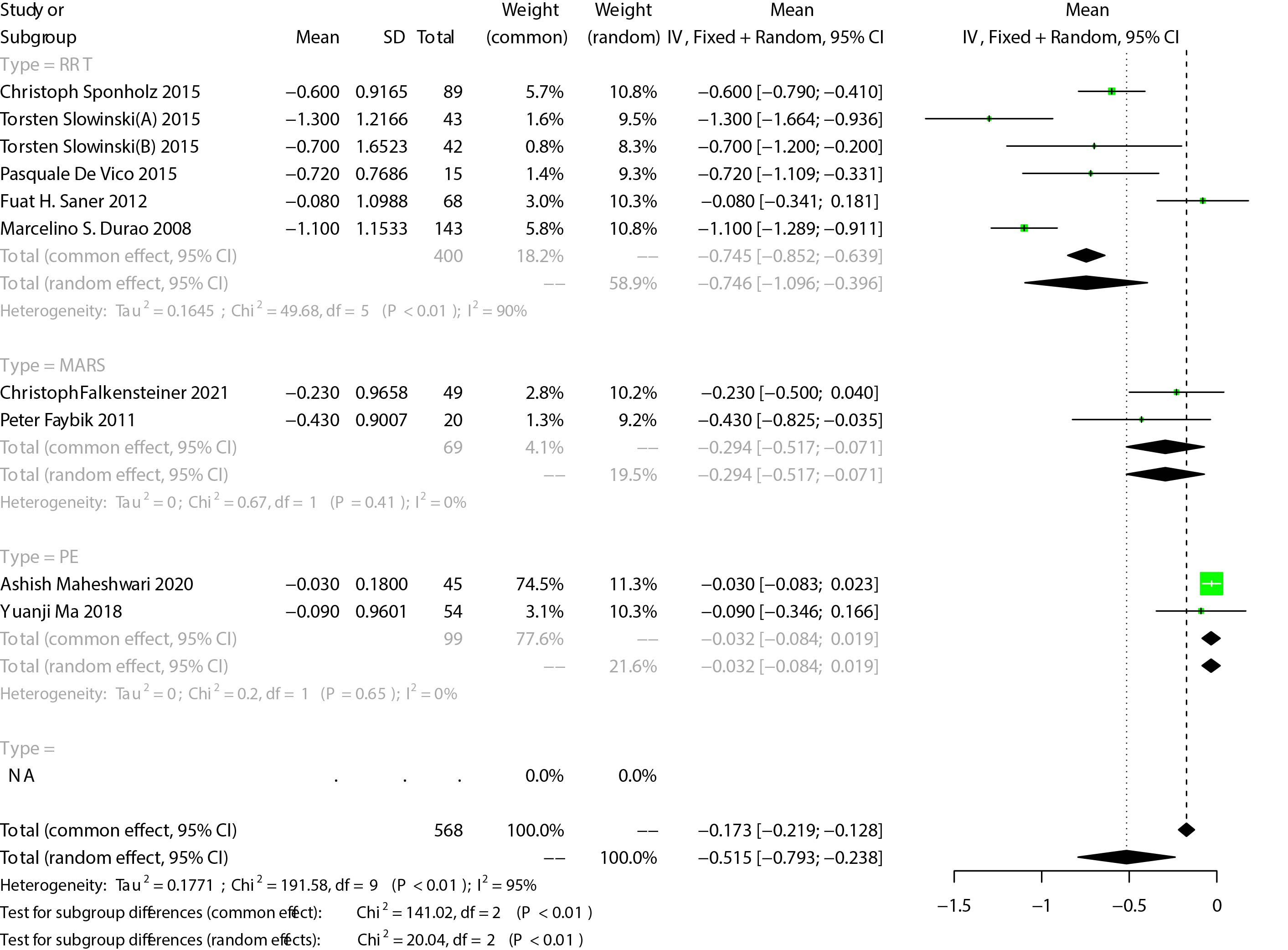

Supplement: Supplementary file 1 [file Presentation_1.zip › 文章补充图/Appendix 3.jpg]

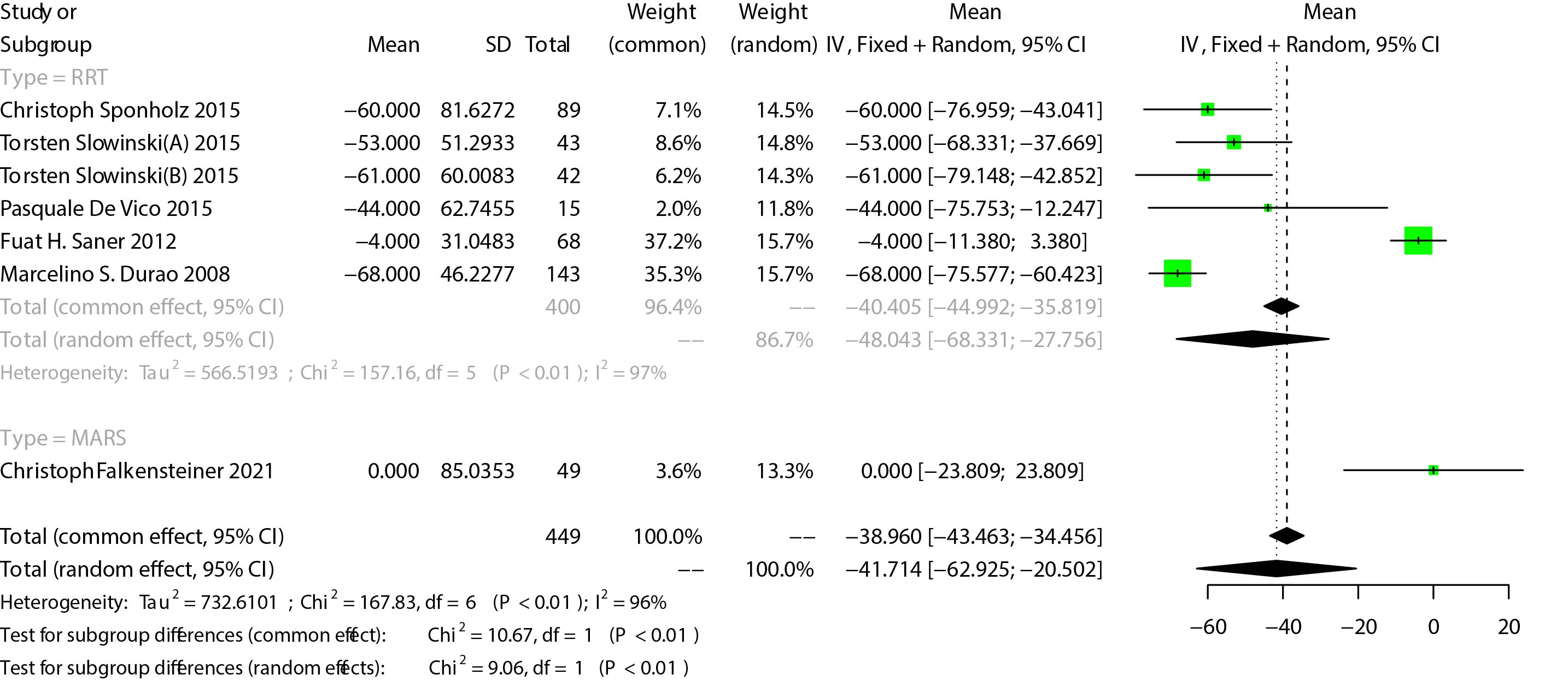

Supplement: Supplementary file 1 [file Presentation_1.zip › 文章补充图/Appendix 4.jpg]

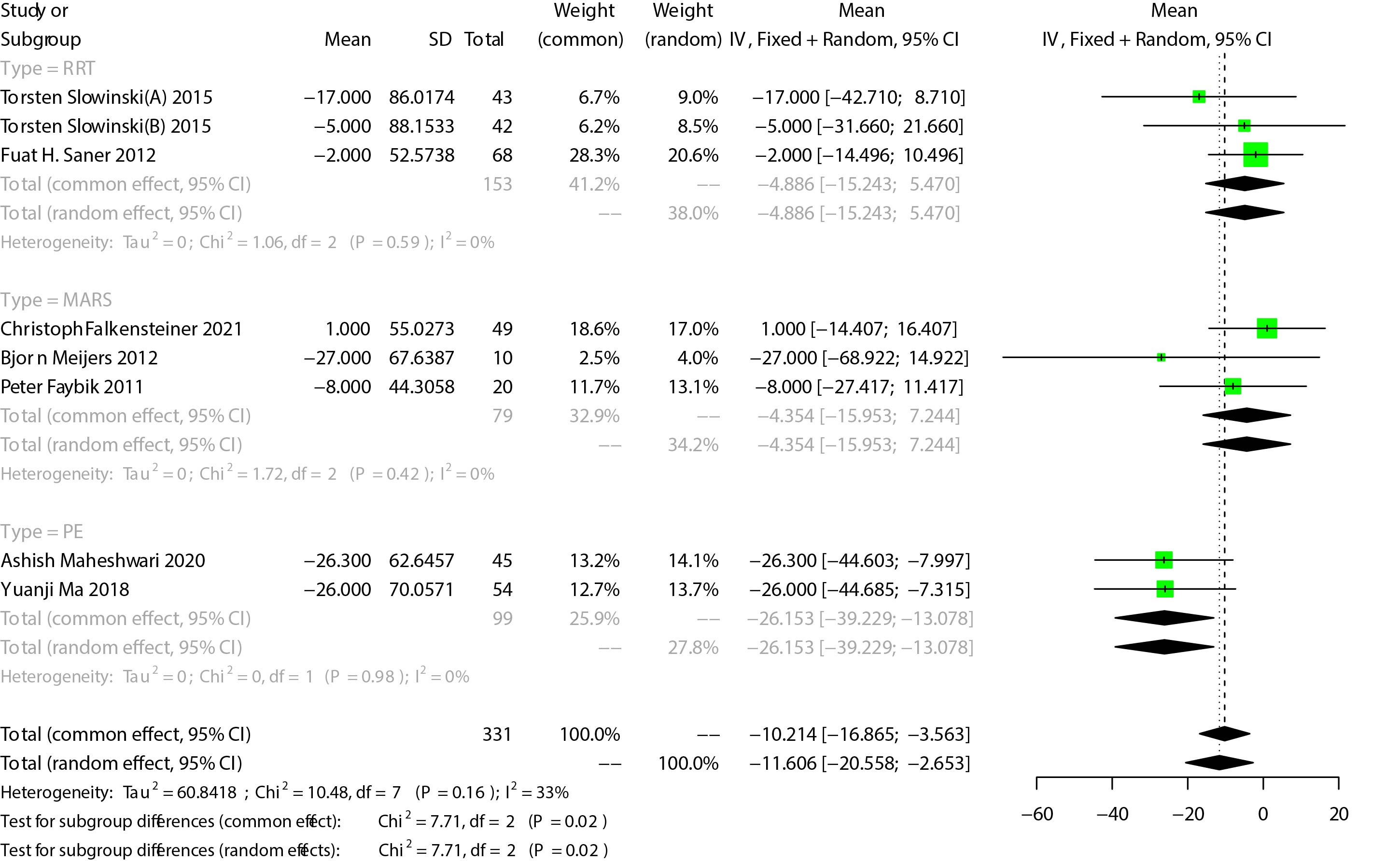

Supplement: Supplementary file 1 [file Presentation_1.zip › 文章补充图/Appendix 5.jpg]

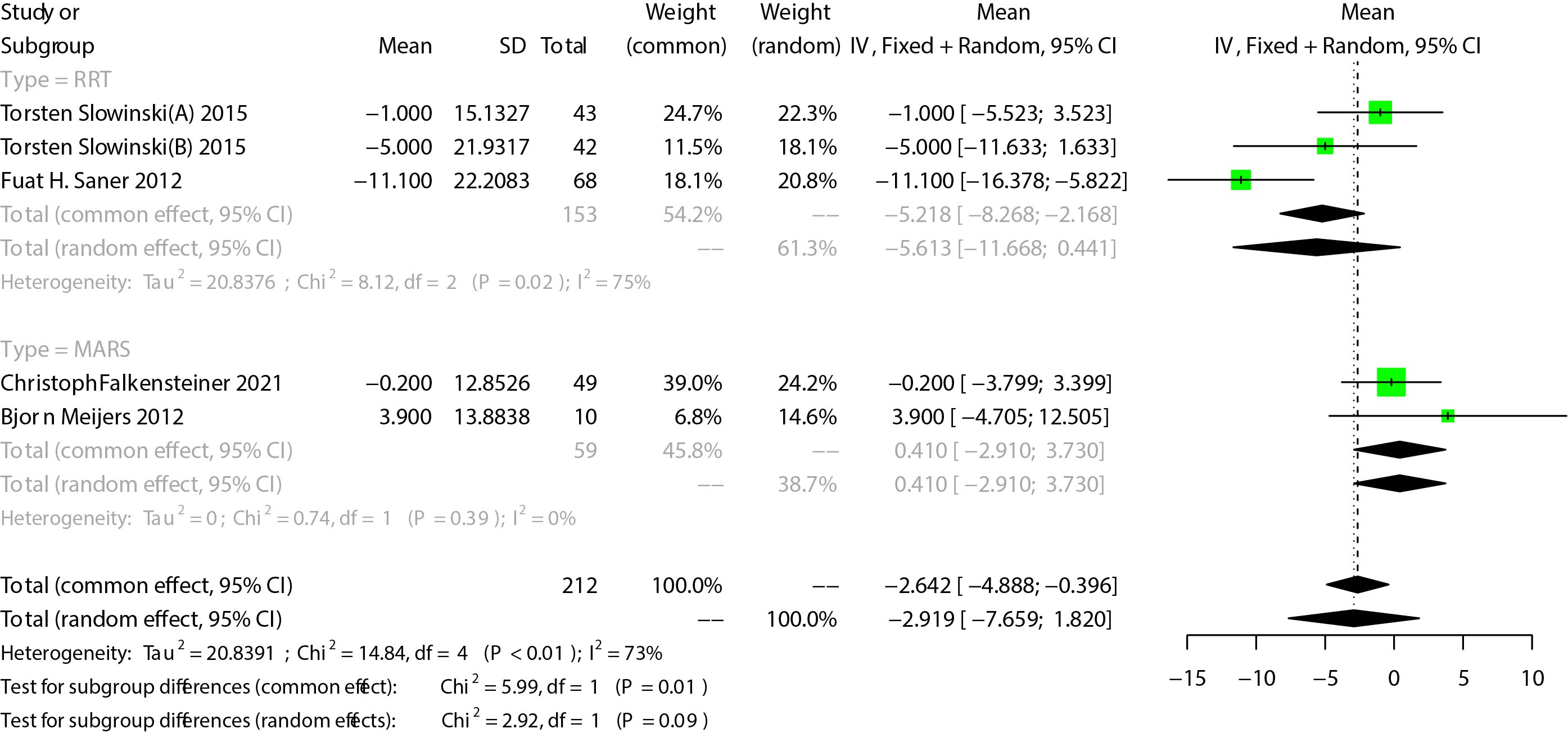

Supplement: Supplementary file 1 [file Presentation_1.zip › 文章补充图/Appendix 6.jpg]

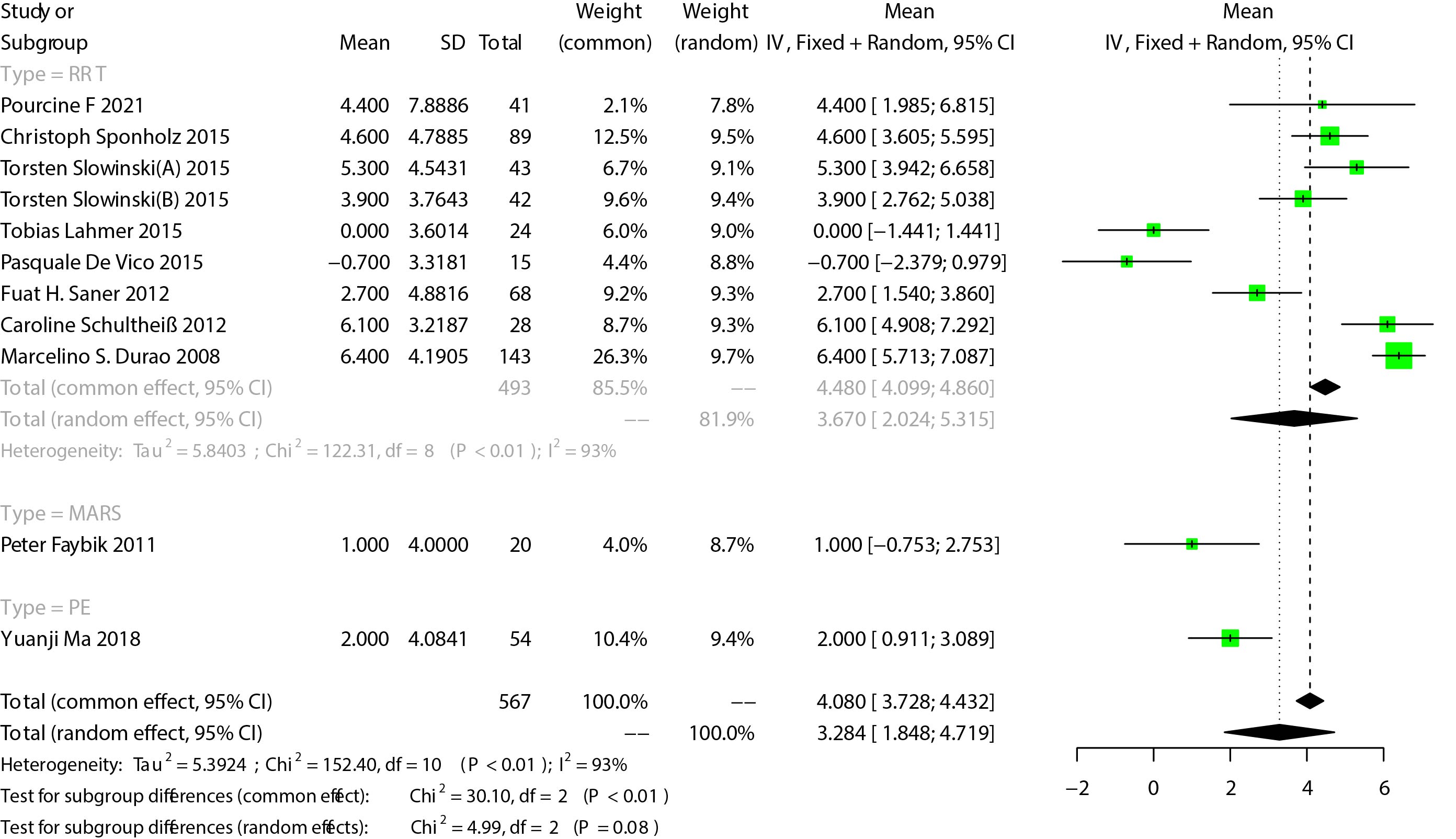

Supplement: Supplementary file 1 [file Presentation_1.zip › 文章补充图/Appendix 7.jpg]

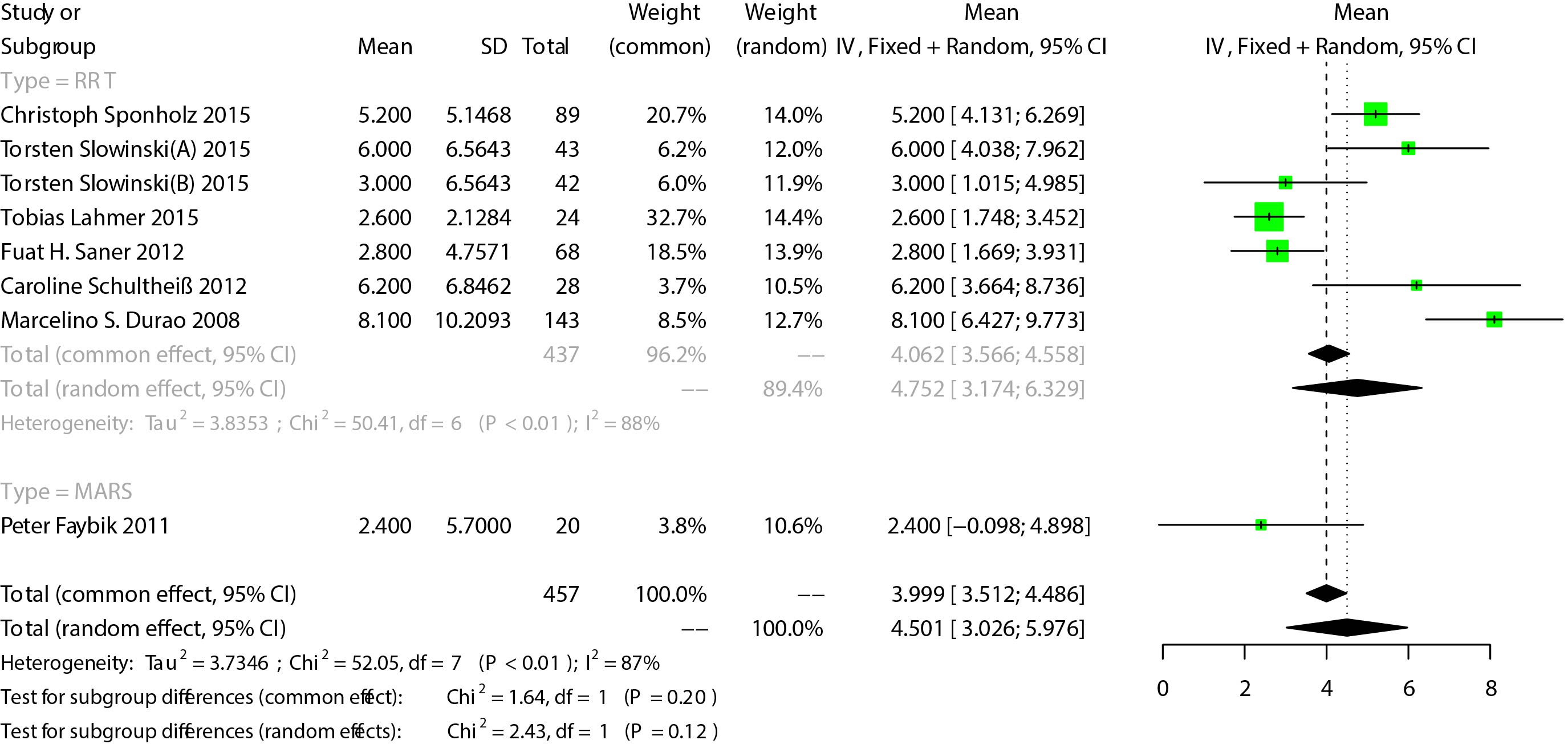

Supplement: Supplementary file 1 [file Presentation_1.zip › 文章补充图/Appendix 8.jpg]

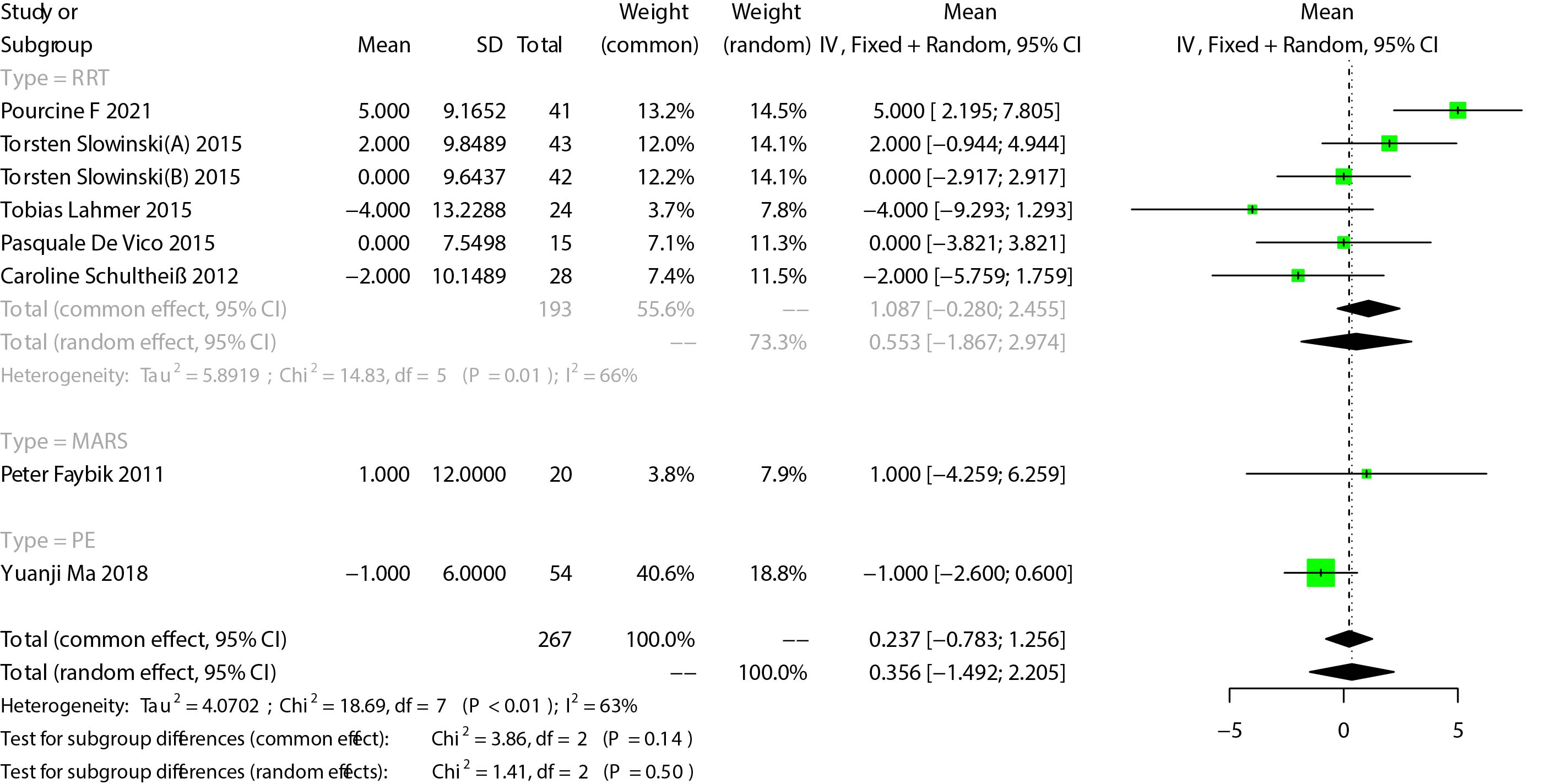

Supplement: Supplementary file 1 [file Presentation_1.zip › 文章补充图/Appendix 9.jpg]
